# Concurrent stimulation of diflufenican biodegradation and changes in the active microbiome in gravel revealed by Total RNA

**DOI:** 10.1128/spectrum.00164-25

**Published:** 2025-08-25

**Authors:** Lea Ellegaard-Jensen, Pedro N. Carvalho, Muhammad Zohaib Anwar, Morten Dencker Schostag, Kai Bester, Carsten Suhr Jacobsen

**Affiliations:** 1 Department of Environmental Science, Aarhus University 307855, Roskilde, Denmark; 2 The Center for Infectious Disease Genomics and One Health, Faculty of Health Sciences, Simon Fraser University 1763, Burnaby, British Columbia, Canada; 3 Department of Biotechnology and Biomedicine, Technical University of Denmark 158981, Kongens Lyngby, Denmark; Ruhr-Universitat Bochum, Bochum, Germany

**Keywords:** biodegradation, pesticides, Total RNA, urban areas, biostimulation, metatranscriptome

## Abstract

The use of slowly degraded pesticides poses a particular problem when these are applied to urban areas such as gravel paths. The urban gravel provides an environment very different from agricultural soils; i.e., it is both lower in carbon and microbial activity. We, therefore, endeavored to stimulate the degradation of the pesticide diflufenican added to urban gravel microcosms amended with dry alfalfa to increase microbial activity. In the present study, alfalfa addition significantly increased the formation of diflufenican’s primary metabolite, 2-[3-(trifluoromethyl)phenoxy]nicotinic acid (AE-B), indicating stimulated biotransformation. The concurrent changes of the active microbial communities within the gravel were explored using shotgun metatranscriptomic sequencing of ribosomal RNA and messenger RNA. Although bacterial taxa remained dominant (87.0%–98.5% relative abundance), the alfalfa treatment led to a 4–5-fold increase in eukaryotic groups, including fungi and microbial grazers. Several microbial taxa potentially involved in the degradation of complex carbon compounds and aromatic pollutants—including Bacteroidetes*,* Verrucomicrobia*,* Sordariomycetes*,* Mortierellales*,* Tremellales, *Sphingopyxis*, and *Phenylobacterium*—increased in relative abundance following alfalfa amendment. Functional gene profiling revealed elevated expression of genes related to microbial activity and biomass production. Genes with potential roles in the breakdown of complex carbon structures (e.g., xylanases/chitin deacetylases) and in the transformation of aromatic compounds (e.g., ring-cleaving dioxygenases) were revealed. We conclude that complex carbon amendments can enhance the microbial activity, promoting the biotransformation of diflufenican in urban gravel environments. These findings provide new insights into the interactions between microbial community dynamics, gene expression profiles, and pesticide biotransformation in non-agricultural matrices.

**IMPORTANCE:**

Pesticides used on urban areas, e.g., gravel paths, are likely to have different effects and fates than when these are used on agricultural soils. Hence, studies into the degradation of pesticides applied to urban matrices are needed. We have previously shown that metabolites of the persistent pesticide diflufenican are even more persistent in urban soils, and it has also previously been shown that these metabolites leach from gravel surfaces. The reasons behind this are that the urban gravel provides an environment very different from agricultural soils; i.e., it is both lower in carbon and microbial activity. In the present study, we, therefore, endeavored to stimulate the degradation of the pesticide diflufenican added to urban gravel microcosms amended with dry alfalfa to increase microbial activity, concurrently studying the changes in the active microbiome by Total RNA-metatranscriptomics.

## INTRODUCTION

Pesticides are applied not only on agricultural fields, but also in urban areas like public or private gardens, gravel paths, terraces, and courtyards (1). It has been reported that private use of herbicides represents 8% of the total usage in the US (1), and that herbicide usage on urban amenity areas accounts for 0.2% and 2.7% in Denmark and The Netherlands, respectively (2). When pesticides are applied to urban gravel areas, their fate in terms of sorption, biodegradation, and leaching is often significantly different from their fate when applied to agricultural soils (3, 4). Diflufenican is an example of an active ingredient in herbicide products applied in both agricultural and urban areas. The literature on pesticide degradation and fate in urban soils is limited, and for diflufenican, it is restricted to only a few papers. We have previously shown that diflufenican, which is used as a weedkiller in urbanized areas, was degraded more slowly in urban gravel (e.g., used on gravel paths) than in agricultural soil, with no mineralization detected and with the continuous formation of the metabolite AE-B (5). It was further shown that diflufenican sorbed much stronger in agricultural soil than in urban gravel, while the sorption of the metabolites AE-0 {2-[3-(trifluoromethyl)phenoxy]-nicotinamide, AE 0542291} and AE-B {2-[3-(trifluoromethyl)phenoxy]nicotinic acid, AE B107137} was weaker than diflufenican in both soil and gravel (5). Together, these findings suggest an increased risk of leaching of diflufenican and AE-B to groundwater when diflufenican is applied on gravel as compared to agricultural soils. Indeed, a leaching experiment with lysimeter columns, containing different gravel types and a sandy arable topsoil, showed that both of diflufenican’s degradation products (AE-0 and AE-B) leached to a greater degree from the gravel columns as compared to the soil columns, resulting in concentrations up to 3.1 µg L^−1^ and 0.5 µg L^−1^ AE-B, respectively, leaching from the pathway gravel and the sandy arable topsoil (4).

The main difference between agricultural soil and urban gravel is the content of organic carbon (3, 6); however, key differences regarding the potential for diflufenican degradation are expected to be related to the microbial activity (although often correlated with organic carbon). As biotic degradation by microorganisms is chiefly responsible for pesticide transformation, previous studies have investigated the effects of pesticide application on soil microbial community and activity (7, 8). A study applying a commercial herbicide containing diflufenican, mesosulfuron-methyl, and iodosulfuron-methyl-sodium in a pot experiment with sandy loam soil found that the dose recommended by the manufacturer did not lead to significant changes in the size of the analyzed microbial populations determined as CFUs of different microbial groups (8). In the same study, however, an effect on the enzyme activity of the microbial community was reported (8). Similarly, another study conducted over 180 days found that diflufenican negatively affected the soil microbial biomass-C (fumigation-extraction) and enzymatic activities in two soil types (7). Together, these studies applying traditional microbial methods signify the importance of investigating the interaction between diflufenican and the active microbiome by modern molecular microbial techniques (e.g., next-generation sequencing).

The slow degradation of diflufenican, with DT₅₀ in lab studies (normalized) of 41.4–318 days and field studies DT₅₀ of 224–621 days (9, 10), has led to studies attempting to stimulate its degradation by the addition of organic materials or biodegrading strains (11–13). One such study added green compost to sandy loam soil without any significant increase in diflufenican degradation compared to the unamended soil (13). Pinto et al. (12) showed that sterile soil inoculated with three fungal strains, *Fusarium oxysporum*, *Paecilomyces variotii,* and *Trichoderma viride* degraded 45%–68% diflufenican during a 120-day experiment. Thus, indicating that fungi may play a key role in degrading diflufenican *in situ*. These previous findings highlight the need for further exploring the entire active microbial community in urban soils, e.g., gravel, and exploration of the potential role that soil fungi play in the transformation of diflufenican. Noticeably, reports on the active microbiome in such systems are lacking in the literature. Furthermore, these studies don’t offer any conclusions regarding the role of metabolic and co-metabolic diflufenican degradation. However, literature on bacterial degradation of other fluorinated organic compounds suggests that this process is typically metabolic (14, 15), whereas fungal species have been shown to degrade fluorinated organic compounds co-metabolically (16, 17).

In this context, the current study focused on stimulating diflufenican biotransformation in an urban gravel, while examining the active microbial community using RNA Illumina shotgun sequencing and metatranscriptomic analysis of the entire active microbial community, i.e., of both prokaryotes and eukaryotes. For this purpose, we applied a complex carbon source in the form of dried alfalfa to the gravel, which has been shown to increase bacterial and fungal soil biomass and activity (18). Alfalfa largely contains cellulose, hemicellulose, and lignin (19), all of which require potent, and often non-specific, microbially produced enzymes for their degradation (20, 21). Hence, this amendment was designed to stimulate the indigenous microbial community and thereby potentially enhance the co-metabolic biotransformation of diflufenican by activating genes related to complex carbon degradation.

The objective of the present study was to stimulate microbial diflufenican transformation in an urban gravel soil matrix by adding a complex carbon source. We hypothesized that this would stimulate, especially, the microorganisms possessing the enzymes to break down complex organic compounds, thus concurrently stimulating diflufenican biotransformation.

## MATERIALS AND METHODS

### Gravel

The type of gravel used in the microcosm experiment was of the brand Slotsgrus (0–11.2 mm, Stenrand Grusgrav, Svebølle, Denmark). This gravel type is typically used in urban areas, where it is used for park trails and in courtyards in public and private areas. The gravel was sieved (<2 mm) and its physicochemical properties characterized previously by Svendsen et al. (5), including its contents of carbon (0.17% C_org_) and nitrogen (0.01% N_org_).

Pellets of dry alfalfa (*Medicago sativa*) (Lucernepiller, dlg, Fredericia, Denmark) were triturated in a blender (JB3010WH, Braun, Braun GmbH, Germany) and mixed into one-half of the gravel at 0.2% (dw) immediately before the microcosm experiment (to simulate the microbial community by the release of carbon and nitrogen from the decaying plant litter), in accordance with previous application procedures (18, 22, 23). The carbon and nitrogen contents of the alfalfa flour were 53.04% C_org_ and 2.06% N_org_. Hence, the contents of carbon and nitrogen in the alfalfa amendment treatment were 0.28% C_org_ and 0.014% N_org_.

### Chemicals

Diflufenican {N-(2,4-difluorophenyl)-2-[3-(trifluoromethyl)phenoxy]-3-pyridinecarboxamide} was purchased from Sigma-Aldrich (Taufkirchen, Germany). Diflufenican metabolites AE B107137 {2-[3-(trifluoromethyl)phenoxy]nicotinic acid (AE-B)} and AE 0542291 {2-[3-(trifluoromethyl)phenoxy]-nicotinamide (AE-0)} were acquired from Apollo Scientific (Cheshire, UK) and Chemieliva Pharmaceutical Co., Ltd. (Chongqing, China), respectively. Internal standard diflufenican-d3 was purchased from Toronto Research Chemicals (Toronto, Canada). Water (LiChrosolv liquid chromatography-mass spectrometry grade), methanol, and acetonitrile (LiChrosolv gradient grade) were acquired from Merck (Darmstadt, Germany). UltraPure phenol:chloroform:isoamyl alcohol in the ratio 25:24:1 used for RNA extraction was from Invitrogen (Thermo Fisher Scientific, Carlsbad, California).

### Microcosm experiment

Setup and sampling of the microcosm experiments were done in a laminar flow bench, and all glassware was pre-sterilized by autoclaving. The microcosms were carried out in brown 60 mL glass jars. These were initially added 0.2 g of sterilized Ottawa sand (50–70 mesh particle size, Sigma-Aldrich, Taufkirchen, Germany) each. Diflufenican dissolved in acetonitrile was added to the sand, where the acetonitrile was allowed to evaporate. Each jar was then added either 25 g (ww) of alfalfa amended gravel or plain gravel and mixed thoroughly with the diflufenican-spiked sand to achieve a final concentration of 330 ng g^−1^. This concentration is based on the recommended application dose of 120 g ha^−1^ (5). Gravel water content was adjusted to pF = 2.2 immediately before use by the addition of sterile MilliQ water. The microcosms were covered with perforated aluminum foil and loosely closed lids to allow access to air. The experiment was incubated in darkness at 16°C, and the water content was maintained by regular addition of sterile MilliQ water to compensate for any observed weight loss due to evaporation from the microcosms.

The microcosms were sampled once a month for chemical analysis over a 6-month period (T0–T5). Here, 1.2 g samples were transferred from triplicate mesocosms of each treatment to 4 mL glass vials and frozen at −18°C for subsequent processing. Likewise, samples for molecular microbial analysis (2 g) were transferred from the triplicate mesocosms of each treatment to sterile, RNase-free 15 mL Nunc tubes and immediately snap-frozen in liquid nitrogen and kept at −80°C until RNA extraction. Samples from day 0 (T0), day 30 (T1), day 59 (T2), and day 119 (T4) were selected for Total RNA library preparation (see section “Total RNA extraction, library prep, and sequencing”).

### Chemical analysis of diflufenican and metabolites

Diflufenican and the metabolites AE-B and AE-0 were extracted from the gravel and analyzed following Albers et al. (4) and Svendsen et al. (5). In brief, the samples were extracted by means of accelerated solvent extraction and quantified by high-performance liquid chromatography coupled with tandem mass spectrometry. Limits of detection were 0.6 ng g^−1^ for diflufenican, 1.5 ng g^−1^ for AE-B, and 0.6 ng g^−1^ for AE-0, while recovery rates were 98%, 76%, and 75%, respectively.

### Total RNA extraction, library prep, and sequencing

RNA was extracted directly from the frozen samples with the addition of 2 mL of G2 DNA/RNA Enhancer (Ampliqon, Odense, Denmark) using the RNeasy PowerSoil Total RNA Kit (Qiagen, København, Denmark) with phenol:chloroform:isoamyl alcohol following the manufacturer’s instructions, except that the RNA was eluted in a final volume of 50 µL instead of 100 µL. DNase treatment was performed using DNase Max (Qiagen) according to the manufacturer’s instructions. The quantity and fragment size of the extracted RNA were determined on a Qubit 4.0 fluorometer (Invitrogen, Eugene, Oregon, US) and Tapestation (Agilent, Santa Clara, CA, USA). The library was prepared using the NEBNext Ultra II Directional RNA Library Prep Kit for Illumina (New England BioLabs, Ipswich, MA, USA) in combination with the NEBNext Multiplex Oligos for Illumina, according to the manufacturer’s protocol. The fragment size of the resulting cDNA libraries was verified by Tapestation, and DNA concentrations were measured on Qubit 4.0. Following the samples were equimolarly pooled, and this final library was sequenced on an Illumina NextSeq 500 (Illumina Inc., San Diego, USA) with a High Output 300 cycles kit.

### Bioinformatics processing

In total, 9.8 × 10^8^ raw reads were obtained and processed through the quality control pipeline, as follows: adapters were clipped using the TrimGalore (v0.6.6) tool; a wrapper script for automating cutadapt (24). FastP (25) was used for quality filtering of reads based on quality (q < 20 in a window of 5 consecutive nts) and length (reads shorter than 60 nts) of the reads.

Prior to further analysis, reads were separated into small subunit (SSU) rRNA, large subunit (LSU) rRNA, and non-rRNA sequences through SortMeRNA (v4.3.1) (26). For the SSU, LSU, and non-rRNA read division across samples, see Table S1. The combined pool of SSU rRNA sequences from all samples was co-assembled into full-length SSU rRNA sequences using MetaRib (27). See Table S1 for parameters other than the default ones. The assembly of rRNA reads was evaluated using QUAST (28). A total of 2.9 × 10^7^ ± 6.9 × 10^6^ reads for each sample were co-assembled into 3,759 contigs. The N50 statistic of the assembly was 1,472 with all 3,759 contigs >1,000 nts. The contigs were taxonomically classified using CREST against the SILVA 128 database (29). SSU rRNA reads were mapped to the contigs using BWA (30), resulting in a table of taxonomically annotated read abundance across samples.

Stacked bar charts of the communities and non-metric multidimensional scaling (NMDS) plots based on Bray-Curtis dissimilarity were created in R (v4.1.1) (31) using the following packages and versions: phyloseq (v1.44.0) (32), ggplot2 (v3.4.2) (33), and tidyverse (v2.0.0) (34). The vegan package (v2.6.4) (35) was used to run Adonis for multivariate analysis of differences between the communities of the treatments.

The combined pool of non-ribosomal sequences from all samples was processed through the CoMW pipeline (36) to assemble complete mRNA sequences and annotate these. For general gene annotation, contigs were aligned against the Md5nr protein database (37) and annotated using eggNOG annotation. For specific annotation of genes related to carbohydrate degradation and biosynthesis, contigs were aligned against CAZy database (38) and annotated against the CAZy hierarchical annotation.

To identify the genes that were significantly differentially expressed between treatments, the DESeq2 (v1.34.0) (39) module of the SARTools pipeline (v. 1.8.1) (40) was deployed in R using parametric mean-variance and independent filtering of false discoveries with the Benjamini–Hochberg procedure (*P* > 0.05) to adjust for type 1 error. DESeq2 was additionally used to test differences between treatments for taxonomic features, and MetacodeR (v. 0.3.8) was used to visualize taxonomic data as a heat tree (41).

## RESULTS

### Diflufenican degradation and metabolite formation

**Fig 1:**
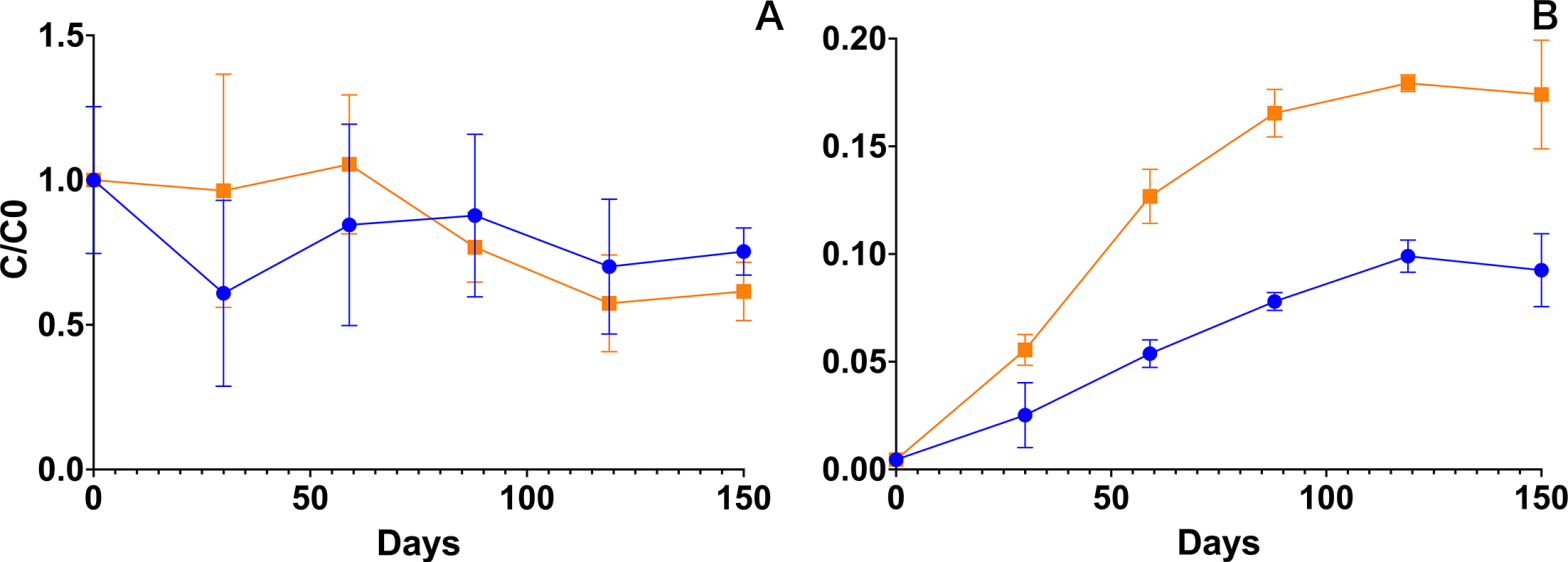
Pesticide residues in the treatments: alfalfa amendment (orange) and control (blue). (**A**) Degradation of diflufenican in the gravel as concentration/initial concentration (C/C0) as a function of time. (**B**) Formation of the metabolite AE-B from diflufenican spiked in gravel. The concentration/initial diflufenican concentration (C/C0) is calculated as the molar concentration of AE-B at time t, divided by the initial molar concentration of diflufenican at time t = 0. All points are averages of three replicate incubations, with the standard deviation shown as an error bar. AE-B stands for 2-[3-(trifluoromethyl)phenoxy]nicotinic acid (EFSA ID—AE B107137).

Samples from six different time points during the 150-day-long experiments were analyzed for diflufenican and its metabolites. Diflufenican was only slowly degraded, and no effect of alfalfa addition on the degradation of diflufenican (Fig. 1A) was found. Accordingly, the final diflufenican concentrations in the alfalfa amended and control treatments of 189.2 (±31.3) ng g^−1^ and 231.8 (±25.1) ng g^−1^, respectively, did not differ significantly (final concentration *t*-test *P* = 0.140).

However, in the alfalfa amended incubations, higher concentrations of the metabolite AE-B were detected compared to the control, as it was more rapidly produced and to a significantly higher final concentration (final concentration *t*-test *p* = 0.001; Fig. 1B), with final concentrations of 38.5 (±5.6) ng g^−1^ and 20.4 (±3.7) ng g^−1^, respectively. In both treatments, AE-B concentration leveled out after 119 days. AE-0 was not detected in any of the samples.

### Total RNA sequencing

Samples from four different time points, day 0 (T0), 30 (T1), 59 (T2), and 119 (T4), were selected for Total RNA sequencing based on the chemical results. In total, 9.83 × 10^8^ raw reads were produced from these 24 samples. Resulting in each sample having 4.05 × 10^7^ ± 7.76 × 10^6^ reads (average ± standard deviation, *n* = 24) following quality filtration. Of these, sorting gave on average 69.8% SSU, 20.4% LSU, and 9.8% non-rRNA (used in the mRNA pipeline). Details are given in Table S1.

### Overall microbial community composition

**Fig 2:**
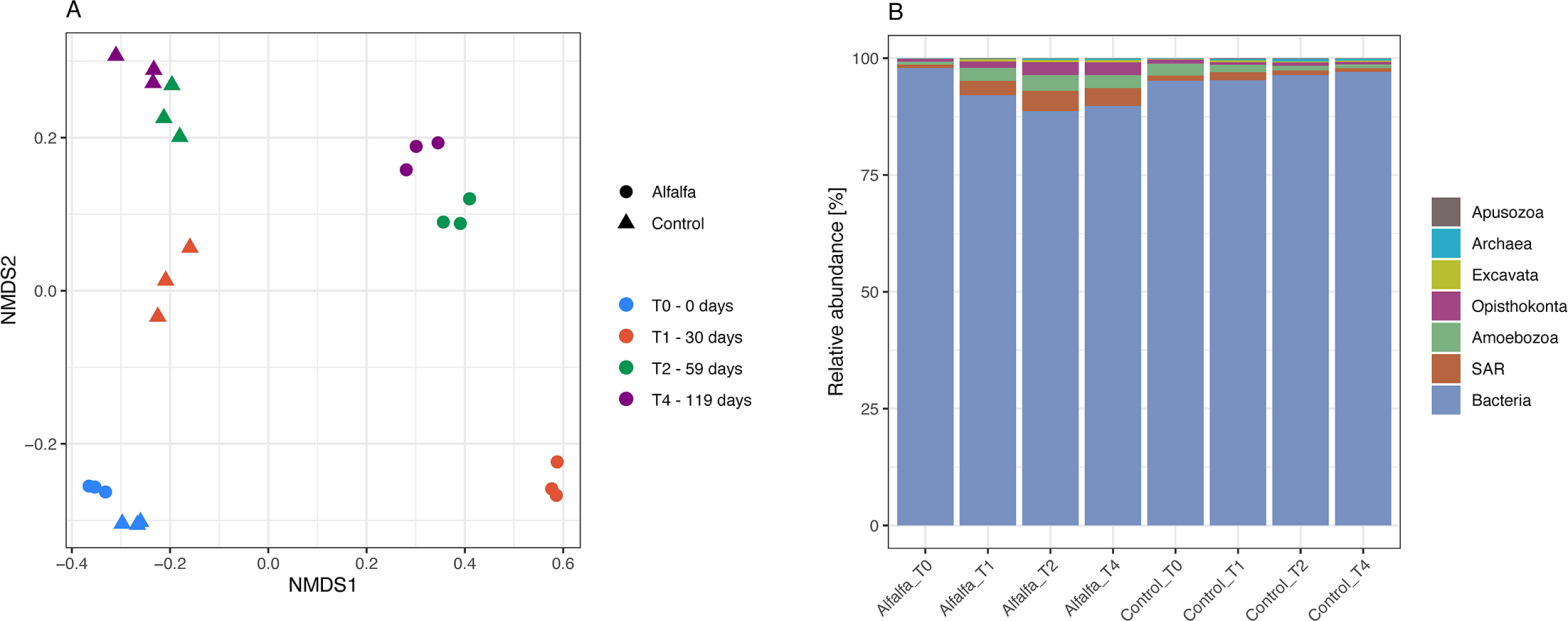
Changes in the overall community profile, based on the small subunit ribosomal RNA, for alfalfa amended and control treatments sampled at day 0, 30 (T1), 59 (T2), and 119 (T4). (**A**) NMDS plot using Bray-Curtis dissimilarity matrices (stress = 0.0297, Shepard plot non-metric *R*^2^ = 0.999) of the active microbial community (rRNA). (B) Composition of the entire active microbial community (rRNA) at the Kingdom or supergroup level.

A total of 3,734 distinct operational taxonomic units (OTUs) were found, of which 3,579 belonged to bacteria, 48 to SAR (Stramenopiles, Alveolates, and Rhizaria) supergroup, 44 to Amoebozoa, 39 to Opisthokonta, 14 to Archaea, 8 to Excavata, and 2 to Apusozoa (Fig. 2). Inspecting further the taxonomy of the eukaryotic groups and resorting these OTUs manually into “fungal” and “other micro-eukaryotes,” 35 OTUs belong to fungal groups, while 106 belong to other eukaryotic groups (i.e., micro-eukaryotes belonging to Amoebozoa, Apusozoa, Excavata, Opisthokonta, and SAR, but excluding fungal groups).

The community composition of the active microorganisms differed significantly between alfalfa amended and control treatments (*P* < 0.001, *R*^2^ = 0.28, *Adonis* [treatment] at OTU level, *n* = 24). The difference appears mainly driven by diverging communities at the three last time points (Fig. 2A), and a significant difference is also found between the community composition of the different sample types, i.e., of specific treatment and sampling time combinations (*P* < 0.001, *R*^2^ = 0.94, *Adonis* [treatment_timepoint]). Although the individual post hoc test was not significant for any two treatments (all *P* > 0.05, *Pairwise Adonis*).

**Fig 3:**
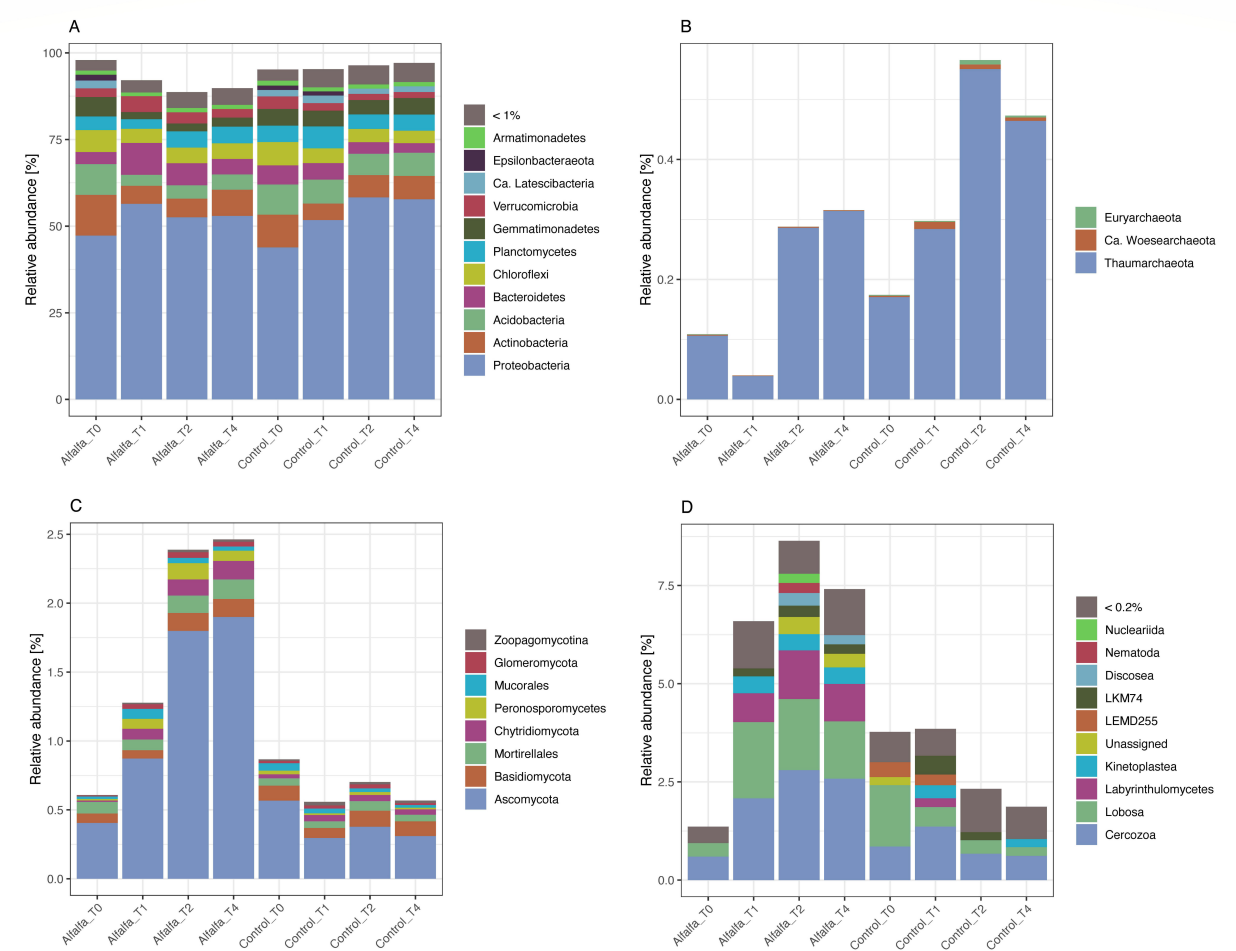
Composition of the active microbial community (rRNA) for alfalfa amended and control treatments sampled at day 0 (T0), 30 (T1), 59 (T2), and 119 (T4) divided into the following groups: (**A**) bacteria, (**B**) archaea, (**C**) fungi, and (**D**) microeukaryotes.

Bacteria had by far the highest relative abundance in all samples, constituting 87.0%–98.5 % of the overall community (Fig. 2B and 3A). For alfalfa amended samples, the relative abundance of bacteria decreased from day 0 with 97.9% ± 0.2% to day 119 with 89.8% ± 0.8%, while the relative abundance of bacteria in the control remained more stable within the range of 94.4%–98.5 %. The relative abundance of archaea was very low, ranging from 0.04%–0.69% of the overall community (Fig. 3B). For alfalfa amended samples, the relative abundance of archaea decreased from day 0 (0.11% ± 0.02%) to day 30 (0.04% ± 0.00%) followed by an increase for days 59 and 119 (0.29% ± 0.05% and 0.32% ± 0.05%, respectively). Whereas for the control, the relative abundance of archaea increased steadily from day 0 (0.17% ± 0.01%) to days 59 and 119 (0.57% ± 0.07% and 0.47% ± 0.20%) (Fig. 3B).

The relative abundance of the combined fungal groups in the overall community ranged from 0.3% to 3.2%. For alfalfa amended samples, the relative abundance of fungi increased gradually from day 0 (0.6% ± 0.1%) to day 119 (2.5% ± 0.2%), whereas the abundance of fungi in the control samples remained relatively stable (0.7% ± 0.2%) (Fig. 3C).

The combined micro-eukaryotic taxa represented 0.9%–9.6 % of the overall microbial community. An almost five times increase in relative abundance was seen in alfalfa amended samples from day 0 with 1.4% ± 0.2% to day 30 with 6.6% ± 0.9%, continuing to a maximum at day 59 with 8.6% ± 0.9% (Fig. 3D). For the control samples, the relative abundance of the micro-eukaryotes was steady from day 0 to 30 then it decreased slightly from day 30 (3.9% ± 0.7%) to days 59 and 119 (2.3% ± 0.2% and 1.9% ± 1.0%) (Fig. 3D).

#### Prokaryotic community composition

**Fig 4:**
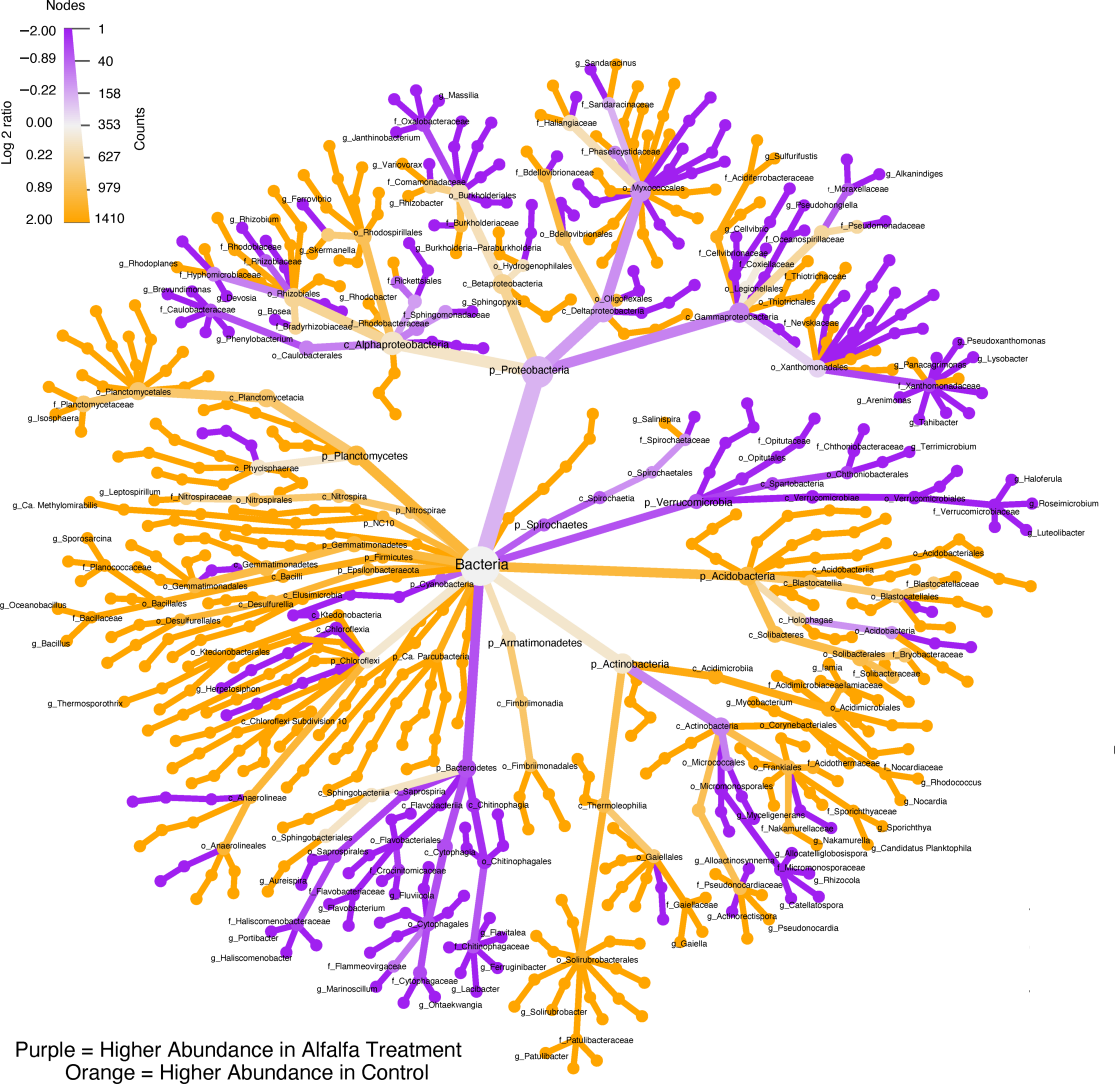
Differential heat tree matrices depicting changes in taxon abundance difference between the alfalfa amended and control treatment in the data subset; bacteria and day 30 (T1). The cladogram shows pairwise comparisons between each treatment, with the color illustrating the log_2_ fold change: a purple node indicates a higher abundance of the taxon in the alfalfa treatment compared to the control. An orange node indicates the opposite. Only taxa with log_2_ fold change >(±)2 at the genus level are shown. All taxon names labeled as “unknown” and in some cases the higher classifications (e.g., class names) are removed for legibility.

The most abundant phyla of the bacterial communities were Proteobacteria > Actinobacteria > Acidobacteria > Bacteroidetes > Chloroflexi > Planctomycetes > Gemmatimonadetes > Verrucomicrobia (Fig. 3A) as observed in order of descending relative abundance across all samples. Of these, a positive effect of alfalfa amendment was found on Bacteroidetes and Verrucomicrobia at days 30 and 59 (Fig. 4; Table S2, *P*_adj_ < 0.05). For Bacteroidetes, this was mainly due to increases in the orders Chitinophagales, Flavobacteriales, Cytophagales, and Saprospirales (Fig. 4; Table S3) of the taxa that could be assigned at the order level. While all the orders within Verrucomicrobia showed significant increases in the alfalfa amended treatment compared to the control at day 30 (Fig. 4; Table S3, *P*_adj_ < 0.05). Within the above-mentioned orders, all genera with log_2_ fold change >(±)2 likewise increased in abundance in the alfalfa treatment at day 30 (Fig. 4).

Oppositely, distinct taxonomic orders within Acidobacteria, Planctomycetes, and Gemmatimonadetes were either shown to be positively or negatively impacted by the alfalfa amendment compared to the control (Fig. 4; Table S3). The Proteobacteria were positively impacted in the alfalfa treatment at day 30 (Fig. 4, *P* < 0.05), but later at day 119, this phylum was negatively impacted in the alfalfa treatment compared to the control (*P*_adj_ < 0.01). However, this phylum contained 70 orders that were affected differently by the treatment (Table S3). Interestingly, the families Sphingomonadaceae and Caulobacteraceae, with, e.g., genera *Sphingopyxis* and *Phenylobacterium*, respectively, increased in abundance in the alfalfa amended treatment compared to the control (Fig. 4). *Phenylobacterium,* represented by a single contig, increased in relative abundance by 300%–1,150% at days 30–119 compared to the control. While *Sphingopyxis,* represented by two contigs, increased in relative abundance by 350%–700% at days 30–119 compared to the control.

For archaea, only three phyla were found. These were Thaumarcheotal > Ca. Woesearchaeota > Euryarchaeota (Fig. 3B). All three phyla were decreased in abundance at days 30–119 in the alfalfa treatment compared to the control (Table S2; all *P*_adj_ < 0.001). At the order level, all taxa were likewise negatively affected, including Methanosarcinales, Nitrososphaerales, and Pacearchaeota (class) (Table S3; *P*_adj_ < 0.05).

#### Eukaryotic community composition

**Fig 5:**
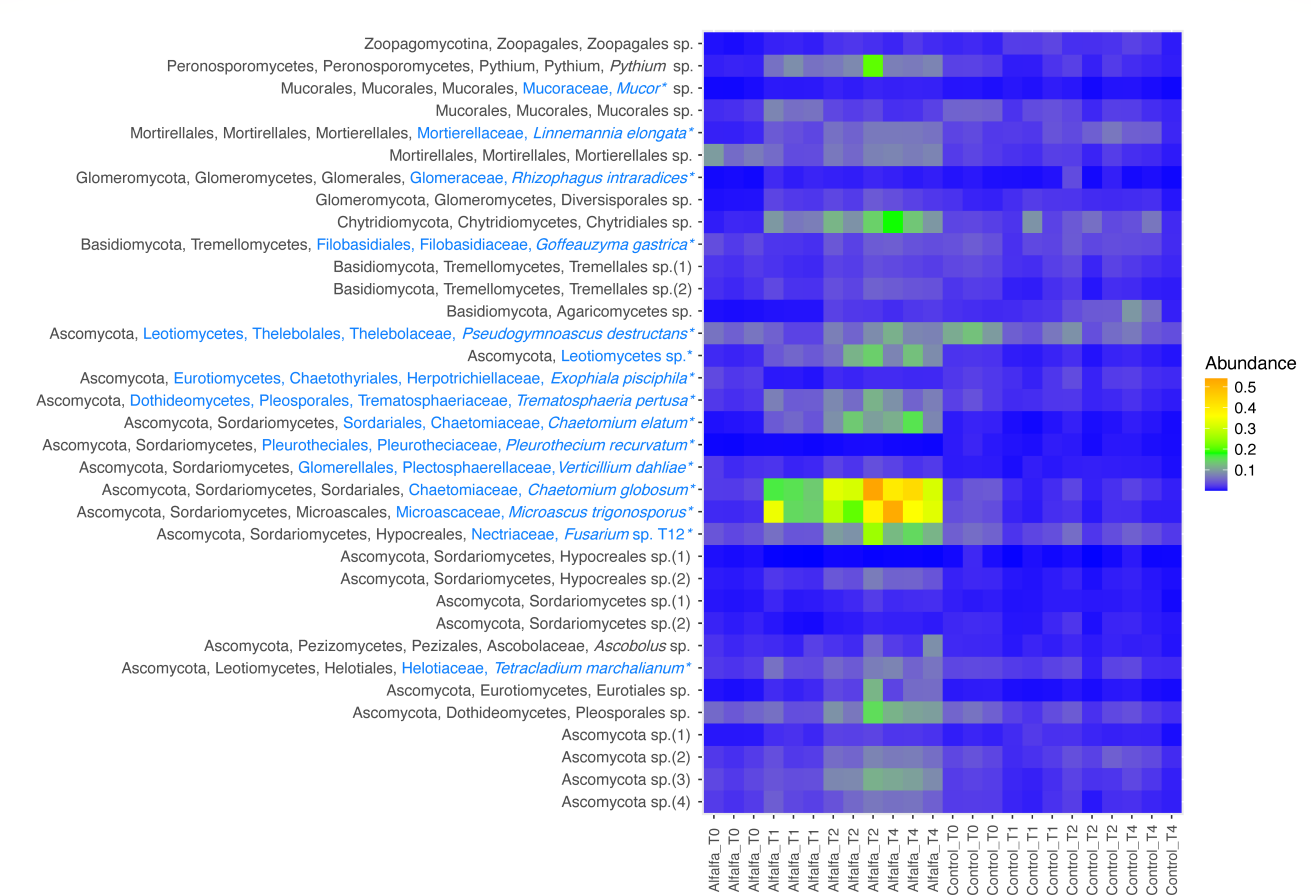
Heatmap of the fungal contig annotations and relative abundance in alfalfa amended and control treatments sampled at day 0 (T0), 30 (T1), 59 (T2), and 119 (T4). Taxonomy is assigned with the SILVA database (black) and the NCBI database (blue; taxonomy with the highest max score by Standard Nucleotide BLAST search [>97% identity] is shown)*.*

The most abundant taxa of the fungal communities were Ascomycota > Basidiomycota > Mortirellales > Chytridiomycota > Peronosporomycetes > Mucorales (Fig. 3C). All these taxonomic groups increased in abundance at day 30 in the alfalfa amended treatment compared to the control (Table S2; *P*_adj_ < 0.05), except Basidiomycota, which remained unaffected (*P*_adj_ > 0.05). At the order level, all taxa within Ascomycota were likewise positively affected at days 30–119 in the alfalfa treatment compared to the control (Table S3; all *P*_adj_ < 0.05). For the Basidiomycota, Tremellales increased in abundance in the alfalfa treatment compared to the control (*P*_adj_ < 0.05), while Agaricomycetes (class) decreased in abundance at days 30 and 119 (Table S3; *P*_adj_ < 0.05). For Mortirellales, Chytridiomycota, Peronosporomycetes, and Mucorales, all orders also increased in abundance at day 30 in the alfalfa treatment compared to the control (Table S3; *P*_adj_ < 0.05). Performing Standard Nucleotide BLAST searches in the NCBI database for the contig sequences belonging to the fungal phyla provided additional taxonomic information, in some cases at species level (Fig. 5). Here, taxa within class Sordariomycetes showed vast increases in relative abundance in the alfalfa amended treatment over time and compared to the control (Fig. 5), with the highest increases seen for *Microascus trigonosporus* and *Chaetomium globosum*.

The remaining eukaryotes—i.e., the micro-eukaryotes within Amoebozoa, Apusozoa, Excavata, Opisthokonta, and SAR, excluding fungal groups—belong to nine taxonomically assigned groups with relative abundance above 0.2% (Fig. 3D). Across all samples, they were observed in the following order of descending relative abundance: Cercozoa > Lobosa > Labyrinthulomycetes > Kinetoplastea > LEMD255 > LKM74 > Discosea > Nematoda > Nucleariida. Eight of these groups were protists, while the last one was the phylum Nematoda (multicellular; Animalia). Cercozoa, Lobosa, Labyrinthulomycetes, Kinetoplastea, Discosea, Nematoda, and Nucleariida all increased in abundance at days 30–119 in the alfalfa amended treatment compared to the control (Table S2; *P*_adj_ < 0.05).

### mRNA—functional genes

**Fig 6:**
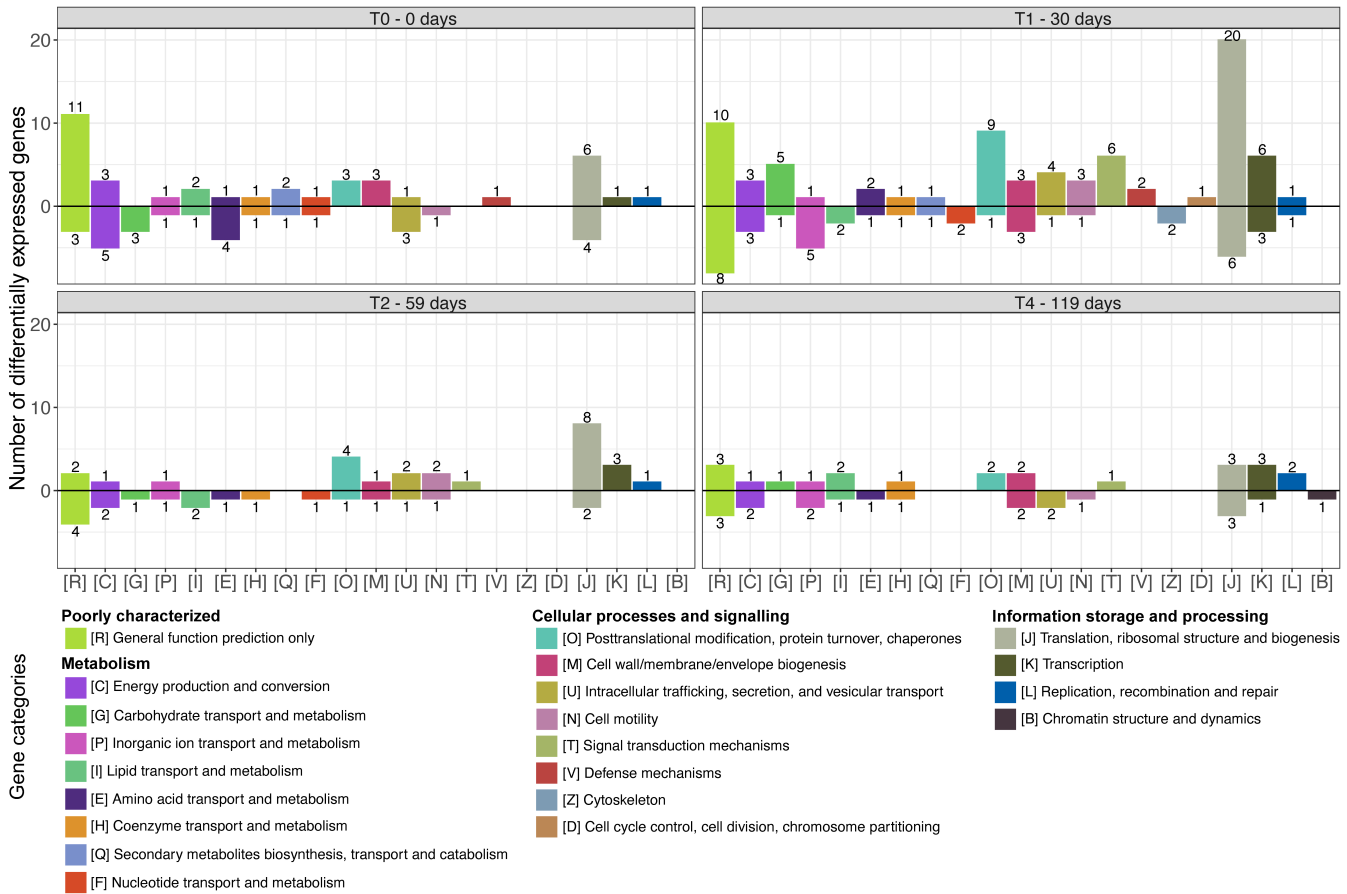
Numbers of differentially expressed genes within functional categories of Md5nr/eggNOG across alfalfa amended and control treatment by pairwise comparisons of gene transcription levels between samples at different incubation times: day 0 (T0), 30 (T1), 59 (T2), and 119 (T4). Increasing and decreasing gene transcription levels are presented above and below the black horizontal zero line, respectively. Digits above/below bars represent the number of differentially expressed genes within a gene category. Gene category “[S] Function unknown” is omitted. See Fig. S1 for all gene categories.

For the genes assigned using the general Md5nr protein database, annotation of the assembled contigs resulted in a combined 1,009 clusters of orthologous groups (COGs) and non-supervised orthologous groups (NOGs) across the samples. Comparing the alfalfa amended and control treatment at the four time points showed that the number of differentially expressed functional genes was highest at day 30 (T1), with 230 genes differentially expressed between the two treatments. At the other three time points, there were approximately 100 differentially expressed genes between the two treatments (Fig. 6; Fig. S1).

A large proportion of the genes differentially expressed between the two treatments falls within the category “Poorly characterized,” e.g., with 108 within “Function unknown” and 18 within “General function prediction only” (Fig. 6; Fig. S1). Within the gene categories of the better-characterized genes, especially “Translation, ribosomal structure, and biogenesis,” “Transcription,” “Signal transduction mechanisms,” and “Post-translational modification, protein turnover, chaperones” showed elevation in the number of COGs/NOGs which are more highly expressed in the alfalfa amended treatment than the control at day 30, compared to the other time points. Likewise, the category “Carbohydrate transport and metabolism,” which could contain genes relevant for the breakdown of organic compounds (e.g., “COG0726 Predicted xylanase/chitin deacetylase”), showed an increase in the number of differentially expressed genes in the alfalfa amended treatment than the control at day 30, compared to the other time points.

The annotation against the CAZy database for genes specific to carbohydrate degradation and biosynthesis yielded 3,504 annotated genes. The differential gene expression between the alfalfa amended and control treatment at the four time points showed that the number was highest at day 30 (T1), with 923 genes differentially expressed between the two treatments. Followed by day 119 and day 59 with 470 and 362 differentially expressed genes, respectively, between the two treatments (Fig. S2).

The highest number of differentially expressed genes at day 30 belonged to the “Carbohydrate-Binding Module” category with 224 upregulated and 107 downregulated, followed by “Glycoside Hydrolases” (up 168, down 146), “Glycosyl Transferases” (up 80, down 129), “Carbohydrate Esterases” (up 20, down 15), “Polysaccharide Lyases” (up 11, down 8), and “Auxiliary Activities” (up 11, down 4). Volcano plots from the SARTools analyses are presented in Fig. S3 and S4. Output from SARTools analyses of mRNA contigs annotated by Md5nr and CAZy is available from https://doi.org/10.5281/zenodo.15574835.

## DISCUSSION

The biotransformation of semi-persistent pesticides applied to urban soils can be correlated to the organic carbon content of the soils (3). The reason for this is that the urban soils, e.g., gravel walkways, are often low in organic carbon and hence also low in microbial activity. In the current study, we therefore added organic carbon to urban gravel to stimulate the indigenous microbial community and thereby enhance the biotransformation of the pesticide diflufenican. The urban gravel used in the current study contained 0.17% C_org_ (5), which is comparable to other urban soils (3), and at least 10 times lower than that of agricultural soil (6). The amendment of 0.2% alfalfa (wt/wt) in the present study is comparable to what is used in other studies and roughly matches agricultural carbon application rates (18, 22, 23). In the present study, we found that alfalfa amendment significantly stimulated the formation of the primary degradation product AE-B, with final concentrations corresponding to 17% and 9% of the added diflufenican for alfalfa and control treatment, respectively. However, this significant effect was only true for the metabolite formation, while the diflufenican removal was similar between treatments due to the large variation of diflufenican results.

Svendsen et al. (5) previously reported that 83% of the initial diflufenican remained in the same type of urban gravel after 150 days. In the present study, 61% and 75% of the spiked diflufenican remained in the alfalfa amended and control treatments after 150 days, respectively. Although this suggests an enhanced diflufenican removal in the alfalfa amended gravel, a clear conclusion cannot be made due to the large variations in the data. A larger standard deviation in diflufenican concentrations in gravel compared to soil due to was previously seen (5). Together, this implies that soil degradation experiment protocols may need to be optimized, e.g., sample mass or longer experimental duration, to accommodate for the higher variation resulting from urban gravel samples.

In comparison, we previously found only 43% of the initial diflufenican present after 150 days of incubation in a sandy agricultural soil (5). Another study found that, on average, 73% of the amount of diflufenican added to two agricultural soils (a sandy loam and a silt-loam) remained after 196 days (42). Hence, diflufenican is considered semi-persistent in most soil environments.

The formation of AE-B from diflufenican in the present study reached a maximum after 119 days, thus indicating that the metabolite might be further degraded. However, we did not detect AE-0 at any time, which is in agreement with our previous experiments in urban gravel (5). Concurrently, we previously found that AE-B could potentially be degraded in the same type of gravel to an uncharacterized metabolite (5), which may impact the formation pattern seen for AE-B. This suggests that further research is needed to determine additional metabolites.

Thus, the alfalfa amendment enhanced the AE-B formation and consequently also the diflufenican biotransformation in the urban gravel used in the present study. This is in line with previous studies showing that the addition of ground lucerne (alfalfa) straw to the fine material of railway ballast (coarse texture and low organic matter content) stimulated microbial activity and led to increased biotransformation of diuron into the metabolites DCPMU and DCPU (43). Another study, however, found no increase in diflufenican degradation when amending the soil with either a spent mushroom substrate or green compost in a field experiment where a herbicide containing diflufenican was applied (13). Although the same study did find that the organic amendments had a stabilizing effect on soil microbial biomass and structure, determined by the phospholipid fatty acid profiles, as well as on microbial dehydrogenase activity following herbicide application (13). Collectively, this supports our initial hypothesis that the addition of a complex carbon source has a stimulating effect on the biotransformation of semi-persistent pesticides in soils or gravel with low carbon contents and low microbial activity, like the urban gravel used in the present study.

The enhanced biotransformation was accompanied by a change in the composition of the active microbial community in the alfalfa amended treatment compared to the control treatment. The Total RNA approach applied in the present study has the strength of revealing changes in the composition of the active microbial community across all domains of life as demonstrated in previous studies investigating the active microbiome of permafrost soil (44), wood ash amended agricultural and forest soil (45), perennial cave ice (46), and moving bed biofilm reactors (47).

In the present study, bacteria dominated the active microbial community in the urban gravel. We saw a decrease in the overall relative abundance of bacteria over time in the alfalfa treatment, whereas the relative abundance of bacteria in the control was quite stable. Despite this decrease in the relative abundance of the overall bacterial community in the alfalfa treatment, we did see an increase in the relative abundance of Bacteroidetes and Verrucomicrobia compared to the control—both of which are known to include species capable of degrading complex carbohydrate-based biomass (48, 49). For Bacteroidetes, a significant increase was found for Chitinophagales, Flavobacteriales, and Saprospirales. Members of these orders are known to encode a large number of CAZymes, including glycoside hydrolases, carbohydrate esterases, and polysaccharide lyases (49), enabling them to degrade either chitin, starch, or cellulose (50, 51). Whether these were involved in the transformation of diflufenican was not possible to elude from the present study, and the literature on pesticide degraders within these phyla appears wanting. Further research is therefore needed to determine possible pesticide-biodegrading species within these phyla. However, we did also find that many other taxa outside the above-mentioned phyla increased in abundance in the alfalfa treatment compared to the control (Fig. 4). Among these were the genera *Sphingopyxis* and *Phenylobacterium*, which have both previously been shown to harbor species capable of degrading aromatic pollutants such as triphenyl phosphate (52) and chloridazon (53). We therefore suggest that species within these genera should be specifically investigated for possible capabilities to degrade diflufenican.

The combined relative abundance of all the fungal taxa increased gradually over time in the alfalfa amended treatment, whereas the abundance of fungi in the control samples remained low and relatively stable over time. Most of the observed increase was due to an increase in Ascomycota, specifically taxa within class Sordariomycetes had a more than 10-fold increase in relative abundance (Fig. 5). Members of this class are present in many ecosystems either as endophytes, pathogens, or saprotrophs involved in decomposition (54). Those standing out with the highest increase are *Microascus trigonosporus* and *Chaetomium globosum* in the alfalfa amended treatment. This is in line with *Chaetomium globosum’s* ability to degrade cellulose-containing substrates (55) and chloroacetanilide herbicide Alachlor (56). For *Microascus trigonosporus,* the closely related *Microascus manginii* has been shown to be capable of degrading pentachlorophenol (57). Furthermore, one of the Sordariomycetes contigs had significant alignments to *Fusarium* sp. T12 (coverage 99%, identity 98.60%) and *F. oxysporum* (coverage 96%, identity 98.76%). *F. oxysporum* has previously been shown to degrade diflufenican during a 120-day experiment (12). This strongly suggests that the alfalfa amendment increases the abundance of this fungal taxon involved in diflufenican degradation.

In addition, other fungi may contribute to the degradation of diflufenican, for instance, species within the orders of Mortierellales and Tremellales, where we saw a significant increase in the relative abundance of these orders in the alfalfa amended treatment compared to the control (Table S3). *Mortierella* species have previously been shown to degrade diuron (58). Similarly, white-rot Tremellales species are capable of degrading complex carbon sources, e.g., lignin and aromatic pollutants, due to their production of polysaccharide- and lignin-degrading enzymes (59). When fungi degrade organic material, they generally use extracellular enzymes to facilitate the breakdown of complex compounds such as lignin and cellulose. These enzymes are non-specific and thus capable of degrading other complex compounds, e.g., pesticides, in soil (60).

Although micro-eukaryotes are not directly involved in biodegradation, they play a key role in shaping microbial communities through their grazing on bacteria (61). In the present study, their overall abundance increased markedly over time in the alfalfa-amended treatment relative to the control, primarily driven by protozoan groups such as Cercozoa, Lobosa, and Labyrinthulomycetes (Fig. 3). Notably, we observed high taxonomic diversity within Cercozoa (34 taxa) and Lobosa (20 taxa), both predominantly bacterivorous (62, 63), likely responding to increased bacterial biomass following alfalfa amendment. Furthermore, Chromadorea (Nematoda) also increased in relative abundance over time in the alfalfa-amended treatment, consistent with previous findings for alfalfa-amended soils (18). The dominant orders, Araeolaimida and Rhabditida, feed on prokaryotes (64), likely contributing to elevated grazing pressure on bacteria and archaea. This suggests that micro-eukaryotes exert top-down control on bacterial populations, potentially obscuring the presence of key bacterial degraders due to intensive grazing.

The functional gene profiles showed that especially genes involved in “Translation, ribosomal structure, and biogenesis” (Md5nr) were more highly expressed in the alfalfa amended treatment than the control at day 30, compared to the other time points. This points to an increase in the activity and production of new microbial biomass in the alfalfa treatment compared to the control, which is expected given the addition of extra carbon to a rather deprived matrix. Furthermore, the application of such a complex carbon source was hypothesized to stimulate the expression of genes coding for potent catabolic enzymes. We found that the gene category “Carbohydrate transport and metabolism,” containing genes for the breakdown of organic compounds, had five genes that had a significantly increased expression in the alfalfa amended treatment at day 30. This included a gene for predicted xylanase/chitin deacetylase, which is well in line with the breakdown of hemicelluloses from alfalfa, given xylanase’s role in hemicellulose degradation (65). Furthermore, the annotation against the CAZy database specific for carbohydrate degradation and biosynthesis genes showed that >200 genes were significantly upregulated for the “Carbohydrate Binding Modules” gene category at day 30. These include genes involved in the binding of cellulose, xylanase, and chitin in line with the results from the Md5nr annotation.

Some genes coding for oxygenases were also found by annotation against Md5nr, but did not show a significantly increased expression. For instance, a gene for aromatic ring-cleaving dioxygenase was equally expressed in alfalfa and control treatments from days 30 to 119. Ring-cleaving dioxygenases are of particular relevance due to their efficacy in the degradation of environmental aromatic pollutants (66)—such as diflufenican. In addition, a gene annotated as Glyoxalase/Bleomycin resistance protein/dioxygenase (general function prediction only) had a great increase in the alfalfa amended treatment with a ∼30 fold change compared to the control at day 0. Finally, several genes belonging to the “Auxiliary Activity” category were annotated as peroxidases by CAZy. These enzymes are known for the degradation of lignin (67) as well as recalcitrant fluorinated pollutants (68). We suggest that further studies should focus on elucidating the role of the abovementioned genes and enzymes in diflufenican degradation.

The present study provides a first glance into the stimulation of the activity of the microbiome in an understudied environmental matrix—the urban gravel—to enhance pesticide degradation. However, the scope of this study was limited by its microcosm design, and hence only included the microbiome native to the gravel. Still, the rRNA revealed an active microbiome, so diverse that unraveling the organisms involved in degradation was only partially possible. The annotated mRNA suffers in part from many biodegradation genes being poorly characterized or altogether missing from the databases. Therefore, we suggest that a combined community complexity reduction and stable isotope probing approach will enable connecting the microbiome and its genes involved in the biodegradation of semi-persistent pesticides.

### Conclusion

We conclude that the amendment with a complex carbon source does enhance biotransformation of the semi-persistent pesticide diflufenican in urban gravel. Concurrently, the Total RNA approach revealed increases in the relative abundance of active microbial groups potentially involved in the degradation of complex carbon sources and diflufenican, such as Bacteroidetes, Verrucomicrobia, Sordariomycetes, Mortierellales, Tremellales, *Sphingopyxis,* and *Phenylobacterium*, as well as increases in the relative abundance of microbial grazers. Finally, the functional gene profile provided insights into the microbial gene expressions—where genes potentially involved in biodegradation of complex carbon sources (e.g., xylanase/chitin deacetylase) and biotransformation of recalcitrant pollutants (e.g., ring-cleaving dioxygenases) are seen as particularly relevant for future research into biodegradation of persistent pesticides.

## Data Availability

The sequence data have been deposited and are publicly available from the NCBI database (BioProject PRJNA1001620). The data on DFF and AE-B concentrations and normalized values are deposited in Zenodo—https://doi.org/10.5281/zenodo.15382982. The counts tables from Total RNA sequencing analyses—rRNA and mRNA, with mRNA annotated by md5nr and CAZy provided as output from SARTools analyses are deposited in Zenodo—https://doi.org/10.5281/zenodo.15574835.

## SUPPLEMENTAL MATERIAL

Supplemental material spectrum.00164-25-s0001.pdf Tables S1 to S3 and Fig. S1 to S4.

ASM does not own the copyrights to Supplemental Material that may be linked to, or accessed through, an article. The authors have granted ASM a non-exclusive, world-wide license to publish the Supplemental Material files. Please contact the corresponding author directly for reuse.
