# Supplementary material for: Concurrent stimulation of diflufenican biodegradation and changes in the active microbiome in gravel revealed by Total RNA
Source: Microbiol Spectr. 2025 Aug 25;13(10):e00164-25. doi: 10.1128/spectrum.00164-25 (PMC12502674; doi:10.1128/spectrum.00164-25)
Supplement: Supplemental material — Tables S1 to S3 and Fig. S1 to S4. [file spectrum.00164-25-s0001.pdf]

Supplementary material

Concurrent stimulation of diflufenican biodegradation and changes in the active microbiome in gravel revealed by Total RNA

Authors

Lea Ellegaard-Jensen[a,*], Pedro N. Carvalho[a], Muhammad Zohaib Anwar[b], Morten Dencker Schostag[c], Kai Bester[a], Carsten Suhr Jacobsen[a]

Institutional Affiliations

[a] *Department of Environmental Science, Aarhus University, Frederiksborgvej 399, Roskilde 4000, Denmark*

[b] *The Center for Infectious Disease Genomics and One Health, Faculty of Health Sciences, Simon Fraser University, 8888 University Dr. W, Burnaby, BC V5A 1S6, Canada*

[c] *Department of Biotechnology and Biomedicine, Technical University of Denmark, Søltofts Plads bldg. 221, DK-2800 Kgs Lyngby Denmark*

* **Corresponding author:** Lea Ellegaard-Jensen, leael@envs.au.dk

Supplementary data

Supplementary Table 1 (part 1/2): Sequence stats during bioinformatic processing.

Alfalfa treatment (%)	Time	1. Quality filtering and sorting into rRNA and mRNA<br>1.1 NextSeq output<br># Raw reads	1.2 TrimGalore<br># Sequences after QC	1.3 SortMeRNA<br># rRNA SSU	# rRNA LSU	# Unaligned (mRNA)
0	T0	36,784,804	36,308,298	25,118,520	7,288,936	3,900,842
0	T0	37,436,962	36,966,622	26,896,918	6,967,254	3,102,450
0	T0	42,163,548	41,792,642	29,043,266	8,497,752	4,251,624
0.2	T0	34,139,688	33,395,778	19,477,330	8,687,238	5,231,210
0.2	T0	38,538,926	38,213,782	27,544,320	5,330,716	5,338,746
0.2	T0	39,199,248	39,006,558	29,473,284	5,804,364	3,728,910
0	T1	34,511,138	34,020,132	20,214,152	9,737,076	4,068,904
0	T1	36,807,416	36,578,136	26,859,846	5,801,854	3,916,436
0	T1	41,835,646	40,862,834	27,840,210	7,677,632	5,344,992
0.2	T1	59,762,880	59,104,628	42,771,614	13,319,554	3,013,460
0.2	T1	58,310,354	57,659,298	42,815,000	12,050,172	2,794,126
0.2	T1	47,247,214	46,751,626	35,594,400	8,942,178	2,215,048
0	T2	35,977,712	35,722,208	21,463,200	11,537,738	2,721,270
0	T2	39,003,624	38,761,864	29,407,430	5,335,744	4,018,690
0	T2	24,239,572	22,981,930	15,510,588	4,242,658	3,228,684
0.2	T2	44,151,126	43,675,492	31,795,050	8,888,324	2,992,118
0.2	T2	42,180,746	41,773,726	29,968,788	8,657,736	3,147,202
0.2	T2	50,029,618	49,497,226	35,478,234	10,831,668	3,187,324
0	T4	41,245,756	40,965,732	30,911,064	6,565,374	3,489,294
0	T4	34,140,556	33,786,814	19,907,550	8,185,358	5,693,906
0	T4	33,570,334	33,302,742	20,848,558	7,746,120	4,708,064
0.2	T4	44,431,560	44,186,390	32,634,600	8,397,504	3,154,286
0.2	T4	42,576,666	42,334,844	30,905,724	8,161,920	3,267,200
0.2	T4	45,081,744	44,789,640	31,781,028	9,420,352	3,588,260
Total sequences		983,366,838	972,438,942	684,260,674	198,075,222	90,103,046

Supplementary Table 1 (part 2/2): Sequence stats during bioinformatic processing.

	2. rRNA assembly	3. mRNA assembly
	2.1 MetaRib assembly[1]	3.1 Trinity assembly
	Assembly stats[2] (complete contig pool)	Assembly stats[3] (complete contig pool)
# contigs	3,759	1,042,590
# contigs (>= 0 bp)	3,759	1,042,590
# contigs (>= 1000 bp)	3,759	12,384
# contigs (>= 5000 bp)	0	89
# contigs (>= 10000 bp)	0	0
Largest contig	1,995	11,518
Total length	5,458,975	322,430,901
Total length (>= 0 bp)	5,458,975	322,430,901
Total length (>= 1000 bp)	5,458,975	18,912,466
Total length (>= 5000 bp)	0	573,642
Total length (>= 10000 bp)	0	56,861
N50	1,472	285
N75	1,400	238
L50	1,798	363,144
L75	2,741	674,004
GC (%)	55.78	56.83
Mapped (%)	72.49	89.28
Avg. coverage depth	11,887	30
Coverage >= 1x (%)	99.35	97.20

More information on the bioinformatic processing can be found in the Materials and Methods section.

[1] from MetaRib.cgf file; [EMIRGE], EM_PARA : --phred33 -l 151 -i 300 -s 75 -a 64 -n 25, EMIRGE references: SILVA_138.1_SSURef_NR99.

[2] Assembly stats produced using QUAST tool of 'all.dedup.filtered'

[3] Assembly stats produced using QUAST tool of 'Trinity'

Supplementary Table 2. DESeq analyses comparing alfalfa and control treatment at phylum level. Log2Fold changes > 0 and < 0 denote increases and decreases, respectively, in the alfalfa treatment compared to the control.

		T0		T1		T2		T4	
	Phylum	log2Fold Change	padj	log2Fold Change	padj	log2Fold Change	padj	log2Fold Change	padj
Bacteria	**Acidobacteria**	0.77	***	-0.75	***	-0.99	***	-1.09	***
	**Actinobacteria**	1.03	**	0.54		-0.56		-0.35	
	Armatimonadetes	0.49	*	0.21		-0.27		-0.54	*
	**Bacteroidetes**	0.04		1.36	***	0.65	**	0.24	
	BRC1	0.78	**	-0.65	*	0.22		0.08	
	Ca. Berkelbacteria	0.61		-1.36	***	-1.35	***	-0.84	*
	Ca. Dependentiae (TM6)	-0.02		-0.12		-0.30		0.03	
	Ca. Hydrogenedentes (NKB19)	1.01	**	-0.69	*	-0.66	**	-0.79	*
	Ca. Latescibacteria (WS3)	1.05	***	-1.77	***	-1.46	***	-1.70	***
	Ca. Microgenomates (OP11)	0.34		-0.17		0.46	**	-0.33	
	Ca. Omnitrophica (OP3)	0.49		-0.66	*	-2.01	***	-2.30	***
	Ca. Parcubacteria	0.18		-1.29	***	-1.24	***	-0.83	**
	Ca. Peregrinibacteria	0.94	**	0.28		-0.35		-0.29	
	Ca. Tectomicrobia	0.40		-2.14	***	-2.65	***	-2.73	***
	Ca. Zixibacteria (RBG-1)	0.76	***	-1.92	***	-2.48	***	-2.74	***
	Chlamydiae	0.49	*	0.85	***	0.80	***	0.63	***
	Chlorobi	0.53	*	0.62	*	0.81	**	0.00	
	**Chloroflexi**	0.65	**	0.35		-0.07		-0.19	
	Cyanobacteria	0.28		1.26	**	0.37		0.76	
	Deinococcus-Thermus	0.92	*	-0.73	*	-1.21	**	-1.81	***
	Elusimicrobia	0.79	**	-1.66	***	-1.20	***	-1.07	***
	Epsilonbacteraeota	1.13	***	-0.83	**	-1.09	***	-1.26	***
	Fibrobacteres	0.44		2.23	***	-1.36	***	-2.84	***
	Firmicutes	1.01	**	-1.13	***	-1.02	**	-0.63	
	Fusobacteria	-0.94		-1.63	*	-2.01	*	-1.61	
	**Gemmatimonadetes**	0.93	***	-0.77	**	-1.22	***	-1.42	***
	Halanaerobiales phylum incertae sedis	0.71	*	-1.49	***	0.64	*	0.53	
	Ignavibacteriae	1.33	***	-1.70	***	-2.13	***	-2.47	***
	Lentisphaerae	0.42		0.69	**	0.68	**	0.60	*
	NC10	1.07	***	-0.81	***	-1.85	***	-2.18	***
	Nitrospirae	0.24		-0.42	*	-0.47	*	-0.73	***
	**Planctomycetes**	0.45	*	-0.79	***	-0.12		-0.40	*
	**Proteobacteria**	0.84	**	0.53	*	-0.45		-0.69	**
	SPAM	0.28		-2.32	***	-2.72	***	-2.57	***
	Spirochaetes	0.19		1.23	***	0.00		-0.38	
	**Verrucomicrobia**	0.20		1.52	***	0.61	**	0.07	
	Unknown	0.75	**	-1.04	***	-0.95	***	-0.75	***
Archaea	**Ca. Woesearchaeota**	0.29		-3.02	***	-2.55	***	-2.75	***
	**Euryarchaeota**	0.07		-3.44	***	-6.68	***	-5.45	***
	**Thaumarchaeota**	0.04		-2.46	***	-1.25	***	-0.94	***
Fungi	**Ascomycota**	0.25		1.97	***	1.97	***	2.25	***
	**Basidiomycota**	0.03		0.12		-0.13		0.01	
	**Chytridiomycota**	-0.76		1.20	**	1.07	*	1.55	***
	Glomeromycota	-0.83	*	1.00	**	0.19		1.06	**
	**Mucorales phylum incertae sedis**	-0.75	*	1.47	***	0.24		1.31	***
	**Mortirellales phylum incertae sedis**	1.42	***	1.14	***	0.57		0.22	
	**Peronosporomycetes phylum incertae sedis**	-0.71		3.07	***	2.34	***	2.32	***
	Zoopagomycotina	-0.87	**	-1.06	***	-0.23		-0.46	
Micro-eukaryotes	Cavosteliida	-0.69		0.64		1.39	***	1.65	***
	Dictyostelia phylum incertae sedis	0.28		5.28	***	6.01	***	6.97	***
	Didymium phylum incertae sedis	0.90		-1.14		1.70	**	0.55	
	**Discosea (Amoebozoa (kingdom))**	-0.92	**	1.16	***	1.52	***	1.57	***
	Gracilipodida	-0.76		1.83	***	2.39	***	2.64	***
	Ischnamoeba phylum incertae sedis	-0.49		1.43	***	0.54		0.82	**
	Lamproderma phylum incertae sedis	-1.85	**	-1.38	*	0.83		-0.96	
	**LEMD255**	-1.05	***	-1.53	***	-0.68	**	-0.71	**
	**LKM74**	-1.09	**	-0.85	**	0.18		0.25	
	**Lobosa**	-1.46	***	2.39	***	2.10	***	2.35	***
	Schizoplasmodiida	-0.96	**	-0.95	**	0.38		0.50	
	Stemonitis phylum incertae sedis	-0.34		-2.00	***	-0.80		-1.13	*
	Unknown Amoebozoa (kingdom) phylum 1	-0.85	**	-0.59	*	-0.91	**	-0.84	**
	Unknown Amoebozoa (kingdom) phylum 2	-2.07	***	2.52	***	2.54	***	3.12	***
	Unknown Amoebozoa (kingdom) phylum 3	-1.03	**	1.00	***	0.96	**	1.03	***
	Apusozoa	-1.12	***	0.41		-1.33	***	-0.76	**
	Bicosoecida phylum incertae sedis	-0.54		0.47		0.52	*	1.08	***
	**Cercozoa**	0.22		1.02	***	1.76	***	1.75	***
	Chrysophyceae phylum incertae sedis	-0.47		3.14	***	2.85	***	3.74	***
	Ciliophora	0.14		1.66	***	0.10		-0.20	
	Heterolobosea	-0.92		2.39	***	2.75	***	2.95	***
	Jakobida	-1.18	***	0.50		1.00	***	1.03	***
	**Kinetoplastea**	-1.29	**	0.78		1.00	*	0.95	*
	**Labyrinthulomycetes phylum incertae sedis**	-0.51		2.13	***	2.51	***	2.71	***
	**Nematoda**	-0.45		4.36	***	3.51	***	4.56	***
	**Nucleariida**	-0.04		4.61	***	5.30	***	4.97	***
	Unknown Rhizaria phylum 1	-1.16	*	-1.71	***	-0.94		0.01	
	Xanthophyceae	-1.19	***	-0.15		0.73	*	1.29	***
	Unknown	-0.40		0.19		1.95	***	2.18	***

*	padj < 0.05
**	padj < 0.01
***	padj < 0.001

Supplementary Table 3 (part 1/4). DESeq analyses comparing alfalfa and control treatment at order level. Log2Fold changes > 0 and < 0 denote increases and decreases, respectively, in the alfalfa treatment compared to the control.

					T0		T1		T2		T4	
	Kingdom	Phylum	Class	Order	log2Fold Change	padj	log2Fold Change	padj	log2Fold Change	padj	log2Fold Change	padj
Bacteria (1/3)	Bacteria	Acidobacteria	Acidobacteria clade RB25	Acidobacteria group 22	0.57	*	-1.90	***	-0.75	***	-0.73	***
	Bacteria	Acidobacteria	Acidobacteria group 11	-	-0.30		-1.08	***	-0.65	***	-0.56	***
	Bacteria	Acidobacteria	Acidobacteria group 12	-	-0.05		-0.71	***	-1.80	***	-2.21	***
	Bacteria	Acidobacteria	Acidobacteria group 13	-	0.27		-1.62	***	-1.47	***	-1.65	***
	Bacteria	Acidobacteria	Acidobacteria group 15	-	-0.02		-2.50	***	-2.77	***	-2.90	***
	Bacteria	Acidobacteria	Acidobacteria group 17	-	0.47	**	-1.14	***	-1.08	***	-1.02	***
	Bacteria	Acidobacteria	Acidobacteria group 18	-	0.44	***	-0.41	***	-0.74	***	-0.61	***
	Bacteria	Acidobacteria	Acidobacteria group 2	-	0.28		-2.27	***	-2.07	***	-2.42	***
	Bacteria	Acidobacteria	Acidobacteria group 5	-	0.29		-1.26	***	-1.02	***	-1.15	***
	Bacteria	Acidobacteria	Acidobacteriia (Acidobacteria group 1)	Acidobacteriales	0.02		-1.75	***	-2.12	***	-2.18	***
	Bacteria	Acidobacteria	Acidobacteriia (Acidobacteria group 1)	Unknown Acidobacteriia order 1	-0.18		-1.86	***	-1.91	***	-1.77	***
	Bacteria	Acidobacteria	AT-s3-28	-	0.28		-0.71	***	-0.58	***	-0.58	***
	Bacteria	Acidobacteria	Blastocatellia (Acidobacteria group 4)	Blastocatellales	0.49	**	0.02		0.01		0.13	
	Bacteria	Acidobacteria	Blastocatellia (Acidobacteria group 4)	Unknown Blastocatellia order 1	0.46		-1.30	***	-0.76	***	-0.55	*
	Bacteria	Acidobacteria	Blastocatellia (Acidobacteria group 4)	Unknown Blastocatellia order 2	-0.38		-2.33	***	-2.01	***	-1.77	***
	Bacteria	Acidobacteria	Holophagae	Acidobacteria group 10	0.17		0.93	***	0.48	*	0.49	*
	Bacteria	Acidobacteria	Holophagae	Acidobacteria group 7a	0.22		-1.71	***	-2.07	***	-1.88	***
	Bacteria	Acidobacteria	Holophagae	Acidobacteria group 7b	0.63		-0.04		-1.36	***	-1.67	***
	Bacteria	Acidobacteria	Holophagae	Holophagales (Acidobacteria group 8)	-0.32		-0.82	*	-1.06	**	-1.96	***
	Bacteria	Acidobacteria	Holophagae	Unknown Holophagae order 1	-0.18		-1.48	***	-1.67	***	-1.69	***
	Bacteria	Acidobacteria	Holophagae	Unknown Holophagae order 2	0.08		0.11		-0.86	***	-1.38	***
	Bacteria	Acidobacteria	Solibacteres (Acidobacteria group 3)	Solibacterales	0.08		-0.37	***	-0.46	***	-0.43	***
	Bacteria	Acidobacteria	Solibacteres (Acidobacteria group 3)	Unknown Solibacteres order 1	0.16		-0.55	***	-0.04		-0.30	*
	Bacteria	Acidobacteria	Solibacteres (Acidobacteria group 3)	Unknown Solibacteres order 2	0.23		0.24		0.50	**	0.85	***
	Bacteria	Acidobacteria	Subgroup 6	-	0.32	**	-0.62	***	-0.60	***	-0.70	***
	Bacteria	Acidobacteria	Unknown Acidobacteria class 1	-	0.45		-0.17		-0.48	*	-0.50	*
	Bacteria	Actinobacteria	Acidimicrobiia	Acidimicrobiales	0.57	*	-0.99	***	-0.89	***	-0.57	*
	Bacteria	Actinobacteria	Acidimicrobiia	Unknown Acidimicrobiia order 1	0.36		0.14		0.09		0.87	**
	Bacteria	Actinobacteria	Acidimicrobiia	Unknown Acidimicrobiia order 2	0.26		-0.16		-0.56	**	-0.29	
	Bacteria	Actinobacteria	Acidimicrobiia	Unknown Acidimicrobiia order 3	-0.03		-0.64	*	-0.29		-0.04	
	Bacteria	Actinobacteria	Actinobacteria (class)	-	0.51		1.13	***	0.56		0.89	**
	Bacteria	Actinobacteria	Actinobacteria (class)	Corynebacteriales	0.29		-1.92	***	-1.56	***	-1.40	***
	Bacteria	Actinobacteria	Actinobacteria (class)	Frankiales	0.38		-0.16		-1.17	***	-0.68	**
	Bacteria	Actinobacteria	Actinobacteria (class)	Kineosporiales	0.91	*	-0.45		-0.21		0.40	
	Bacteria	Actinobacteria	Actinobacteria (class)	Micrococcales	0.88	*	1.31	***	0.61		0.85	*
	Bacteria	Actinobacteria	Actinobacteria (class)	Micromonosporales	0.16		4.07	***	2.38	***	2.77	***
	Bacteria	Actinobacteria	Actinobacteria (class)	Propionibacteriales	0.74	*	0.67	*	0.22		0.82	**
	Bacteria	Actinobacteria	Actinobacteria (class)	Pseudonocardiales	0.17		0.14		-0.95	**	-0.39	
	Bacteria	Actinobacteria	Actinobacteria (class)	Streptomycetales	0.48		0.50		0.83	**	1.44	***
	Bacteria	Actinobacteria	Actinobacteria (class)	Streptosporangiales	-0.17		-1.86	***	-1.93	***	-1.47	***
	Bacteria	Actinobacteria	Actinobacteria (class)	Unknown Actinobacteria (class) order 1	0.58	*	0.10		-0.31		-0.25	
	Bacteria	Actinobacteria	Actinobacteria (class)	Unknown Actinobacteria (class) order 2	0.75	*	1.26	***	1.09	***	1.78	***
	Bacteria	Actinobacteria	Actinobacteria (class)	Unknown Actinobacteria (class) order 3	0.30	**	-0.76	***	-0.42	***	-0.33	***
	Bacteria	Actinobacteria	MB-A2-108	-	0.28		-1.50	***	-1.98	***	-2.08	***
	Bacteria	Actinobacteria	Thermoleophilia	Gaiellales	0.43	*	0.08		-0.85	***	-0.70	***
	Bacteria	Actinobacteria	Thermoleophilia	Solirubrobacterales	0.82	**	-0.91	***	-1.39	***	-1.47	***
	Bacteria	Actinobacteria	Thermoleophilia	Unknown Thermoleophilia order 1	0.58	*	-0.93	***	-1.72	***	-1.41	***
	Bacteria	Actinobacteria	Thermoleophilia	Unknown Thermoleophilia order 2	0.54	*	-2.21	***	-1.67	***	-1.37	***
	Bacteria	Armatimonadetes	Armatimonadetes Group 4	-	0.00		-0.04		-0.40	**	-0.58	***
	Bacteria	Armatimonadetes	Armatimonadia	Armatimonadales	0.22		1.60	***	1.13	***	0.94	***
	Bacteria	Armatimonadetes	Armatimonadia	Unknown Armatimonadia order 1	0.26		1.51	***	1.13	***	1.04	***
	Bacteria	Armatimonadetes	Chthonomonadetes	Chthonomonadales	0.19		-0.71	***	-0.59	***	-0.74	***
	Bacteria	Armatimonadetes	Chthonomonadetes	Unknown Chthonomonadetes order 1	0.15		-0.61	***	-0.31		-0.36	*
	Bacteria	Armatimonadetes	Fimbriimonadia	Fimbriimonadales	-0.10		-0.14		-0.29		-0.53	**
	Bacteria	Armatimonadetes	Fimbriimonadia	Unknown Fimbriimonadia order 1	-0.14		1.63	***	0.12		-0.02	
	Bacteria	Bacteroidetes	Bacteroidetes VC2.1 Bac22	-	-0.26		-0.20		0.72	*	0.52	
	Bacteria	Bacteroidetes	Bacteroidia	Bacteroidales	-0.78		-0.71		-4.04	***	-3.52	***
	Bacteria	Bacteroidetes	Bacteroidia	Unknown Bacteroidia order 1	0.33		-1.48	***	-1.17	***	-0.93	***
	Bacteria	Bacteroidetes	Chitinophagia	Chitinophagales	-0.05		2.13	***	2.15	***	1.76	***
	Bacteria	Bacteroidetes	Cytophagia	Cytophagales	-0.84	***	1.53	***	0.46	*	0.02	
	Bacteria	Bacteroidetes	Cytophagia	Unknown Cytophagia order 1	-0.92	***	1.61	***	0.36		-0.24	
	Bacteria	Bacteroidetes	Cytophagia	Unknown Cytophagia order 2	-0.60	**	1.21	***	-0.25		-0.65	***
	Bacteria	Bacteroidetes	Cytophagia	Unknown Cytophagia order 3	0.29		2.09	***	0.64	**	-0.12	
	Bacteria	Bacteroidetes	Flavobacteriia	Flavobacteriales	0.37		2.79	***	2.02	***	1.64	***
	Bacteria	Bacteroidetes	Flavobacteriia	Unknown Flavobacteriia order 1	0.06		1.85	***	1.24	***	1.20	***
	Bacteria	Bacteroidetes	Saprospiria	Saprospirales	0.14		1.80	***	2.26	***	2.10	***
	Bacteria	Bacteroidetes	SM1A07	-	-0.58		0.39		-1.35	*	-2.63	***
	Bacteria	Bacteroidetes	Sphingobacteriia	Sphingobacteriales	-0.29		0.24		-0.23		-0.63	
	Bacteria	Bacteroidetes	Sphingobacteriia	Unknown Sphingobacteriia order 1	-0.14		-3.44	***	-2.36	***	-2.08	***
	Bacteria	BRC1	-	-	0.28		-0.48		0.59	*	0.46	
	Bacteria	Ca. Berkelbacteria	-	-	0.11		-1.18	***	-0.97	***	-0.42	
	Bacteria	Ca. Dependentiae (TM6)	-	-	-0.51	**	0.03		0.07		0.43	**
	Bacteria	Ca. Hydrogenedentes (NKB19)	-	-	0.51		-0.53	*	-0.28		-0.26	
	Bacteria	Ca. Latescibacteria (WS3)	-	-	0.55	*	-1.61	***	-1.09	***	-1.23	***
	Bacteria	Ca. Microgenomates (OP11)	Candidatus Levybacteria	-	-0.10		-1.31	***	-1.45	***	-1.43	***
	Bacteria	Ca. Microgenomates (OP11)	Candidatus Pacebacteria	-	-0.37		1.98	***	3.11	***	2.40	***
	Bacteria	Ca. Microgenomates (OP11)	Candidatus Roizmanbacteria	-	-0.27		-0.18		-0.26		-0.06	
	Bacteria	Ca. Omnitrophica (OP3)	-	-	-0.01		-0.47		-1.82	***	-2.07	***
	Bacteria	Ca. Omnitrophica (OP3)	Ca. Omnitrophus class	Ca. Omnitrophus order	0.00		-0.55		0.42		-0.59	
	Bacteria	Ca. Parcubacteria	-	-	-0.49	*	0.05		-0.55	**	-0.22	
	Bacteria	Ca. Parcubacteria	Candidatus Adlerbacteria	-	-0.23		0.57	**	-0.41	*	-0.35	
	Bacteria	Ca. Parcubacteria	Candidatus Azambacteria	-	-0.75	**	-0.56	*	-0.55	*	-0.31	
	Bacteria	Ca. Parcubacteria	Candidatus Jorgensenbacteria	-	-0.56		-1.54	***	-2.52	***	-1.74	***
	Bacteria	Ca. Parcubacteria	Candidatus Kuenenbacteria	-	0.06		-2.42	***	-1.69	***	-1.31	***
	Bacteria	Ca. Parcubacteria	Candidatus Magasanikbacteria	-	-0.06		-1.78	***	0.08		0.39	
	Bacteria	Ca. Parcubacteria	Candidatus Nomurabacteria	-	-0.97	*	-0.89	*	0.05		0.89	*
	Bacteria	Ca. Parcubacteria	Unknown Ca. Parcubacteria class 1	-	0.05		-3.35	***	-2.43	***	-1.76	***
	Bacteria	Ca. Peregrinibacteria	Candidatus Peribacteria	-	0.44		0.46		0.03		0.10	
	Bacteria	Ca. Tectomicrobia	-	-	-0.09		-1.96	***	-2.26	***	-2.30	***
	Bacteria	Ca. Zixibacteria (RBG-1)	-	-	0.26		-1.74	***	-2.11	***	-2.34	***

*	padj < 0.05
**	padj < 0.01
***	padj < 0.001

Supplementary Table 3 (part 2/4). DESeq analyses comparing alfalfa and control treatment at order level. Log2Fold changes > 0 and < 0 denote increases and decreases, respectively, in the alfalfa treatment compared to the control.

					T0		T1		T2		T4	
	Kingdom	Phylum	Class	Order	log2Fold Change	padj	log2Fold Change	padj	log2Fold Change	padj	log2Fold Change	padj
Bacteria (2/3)	Bacteria	Chlamydiae	Chlamydiia	Chlamydiales	0.00		1.00	***	1.17	***	1.01	***
	Bacteria	Chlorobi	Chlorobia	Chlorobiales	0.03		0.79	***	1.18	***	0.46	*
	Bacteria	Chloroflexi	1-20	-	-0.37		-0.40		-0.16		0.06	
	Bacteria	Chloroflexi	Anaerolineae	Anaerolineales	0.19		-0.71	***	-0.04		0.26	
	Bacteria	Chloroflexi	Anaerolineae	Unknown Anaerolineae order 1	-0.06		-1.62	***	-0.93	***	-1.08	***
	Bacteria	Chloroflexi	Anaerolineae	Unknown Anaerolineae order 2	-0.11		3.47	***	0.35		-0.33	
	Bacteria	Chloroflexi	Anaerolineae	Unknown Anaerolineae order 3	0.27		-0.81	***	-0.21		0.37	
	Bacteria	Chloroflexi	Anaerolineae	Unknown Anaerolineae order 4	0.50	***	-0.29	*	-0.04		-0.26	
	Bacteria	Chloroflexi	Ardenticatenia	-	-0.64		0.06		-0.05		-0.92	*
	Bacteria	Chloroflexi	Ardenticatenia	Ardenticatenales	-0.42		0.26		1.09	***	2.01	***
	Bacteria	Chloroflexi	Caldilineae	Caldilineales	-0.42		-0.39		-0.08		0.22	
	Bacteria	Chloroflexi	Chloroflexi Subdivision 10	Gitt-GS-136	0.77	***	-1.63	***	-1.32	***	-0.94	***
	Bacteria	Chloroflexi	Chloroflexi Subdivision 10	KD4-96	0.53	***	-1.32	***	-1.19	***	-0.94	***
	Bacteria	Chloroflexi	Chloroflexi Subdivision 10	P2-11E	0.76	***	-2.50	***	-2.06	***	-2.05	***
	Bacteria	Chloroflexi	Chloroflexi Subdivision 10	Unknown Chloroflexi order 1	-0.07		-1.52	***	-1.05	***	-1.25	***
	Bacteria	Chloroflexi	Chloroflexi Subdivision 11	-	0.12		-1.59	***	-1.20	***	-1.21	***
	Bacteria	Chloroflexi	Chloroflexi Subdivision 2	-	-0.35		3.16	***	2.60	***	2.75	***
	Bacteria	Chloroflexi	Chloroflexi Subdivision 5 (SAR202)	-	0.94	***	-2.41	***	-2.47	***	-2.35	***
	Bacteria	Chloroflexi	Chloroflexi Subdivision 8 (TK10)	-	0.23		-1.49	***	-1.29	***	-1.16	***
	Bacteria	Chloroflexi	Chloroflexia	Chloroflexales	0.10		-0.77	***	-0.04		0.00	
	Bacteria	Chloroflexi	Chloroflexia	Herpetosiphonales	0.59	*	5.61	***	6.58	***	6.88	***
	Bacteria	Chloroflexi	Chloroflexia	Kallotenuales	0.00		-0.93	***	-0.81	***	-0.63	**
	Bacteria	Chloroflexi	JG30-KF-CM66	-	0.38		-1.62	***	-1.30	***	-1.06	***
	Bacteria	Chloroflexi	Ktedonobacteria	Ktedonobacterales	-0.84	*	-2.15	***	-1.26	***	-0.62	
	Bacteria	Chloroflexi	NLS2-31	-	0.18		-0.79	**	-0.57		-0.11	
	Bacteria	Chloroflexi	S085	-	0.23		-1.18	***	-0.94	***	-0.72	***
	Bacteria	Chloroflexi	SBR2076	-	-0.07		-0.58	***	0.42	***	1.03	***
	Bacteria	Chloroflexi	Thermomicrobia	JG30-KF-CM45	-0.20		-0.57		-0.54		0.03	
	Bacteria	Cyanobacteria	Ca. Melainabacteria	Vampirovibrionales	0.70		4.87	***	3.82	***	5.55	***
	Bacteria	Cyanobacteria	Melainabacteria	Obscuribacterales	0.44		-0.90	*	0.89	*	1.09	*
	Bacteria	Cyanobacteria	ML635J-21	-	-0.31		1.53	**	0.61		0.90	
	Bacteria	Deinococcus-Thermus	Deinococci	Deinococcales	0.43		-0.54	*	-0.84	**	-1.42	***
	Bacteria	Elusimicrobia	Elusimicrobia (class)	FCPU453	0.54		-1.65	***	-2.42	***	-1.62	***
	Bacteria	Elusimicrobia	Elusimicrobia (class)	Lineage IIa	0.25		-0.19		-0.04		0.19	
	Bacteria	Elusimicrobia	Elusimicrobia (class)	Lineage IIb	-0.10		-0.13		0.16		-0.25	
	Bacteria	Elusimicrobia	Elusimicrobia (class)	Lineage IV	0.32		-1.77	***	-0.43		-0.41	
	Bacteria	Elusimicrobia	Elusimicrobia (class)	MVP-88	0.48	*	-0.94	***	-0.72	***	-0.78	***
	Bacteria	Elusimicrobia	Elusimicrobia (class)	Unknown Elusimicrobia (class) order 1	-0.01		-1.40	***	0.16		0.72	***
	Bacteria	Epsilonbacteraeota	Desulfurellia	Desulfurellales	0.63	***	-0.66	***	-0.71	***	-0.78	***
	Bacteria	Epsilonbacteraeota	Desulfurellia	Unknown Desulfurellia order 1	0.46	**	-1.50	***	-1.05	***	-0.97	***
	Bacteria	Fibrobacteres	Fibrobacteria	Fibrobacterales	-0.06		2.41	***	-1.00	**	-2.50	***
	Bacteria	Firmicutes	Bacilli	Bacillales	0.38		-0.96	**	-0.64	*	-0.17	
	Bacteria	Firmicutes	Bacilli	Lactobacillales	6.01	***	-0.06		-3.28	***	-4.48	***
	Bacteria	Fusobacteria	Fusobacteriia	Fusobacteriales	-1.46		-1.43		-1.59	*	-1.12	
	Bacteria	Gemmatimonadetes	AKAU4049	-	0.55		0.24		-1.07	***	-2.19	***
	Bacteria	Gemmatimonadetes	BD2-11 terrestrial group	-	0.37	*	-1.21	***	-0.74	***	-0.97	***
	Bacteria	Gemmatimonadetes	Gemmatimonadetes (class)	Gemmatimonadales	0.44	*	-0.52	**	-0.87	***	-0.95	***
	Bacteria	Gemmatimonadetes	Gemmatimonadetes (class)	Unknown Gemmatimonadetes (class) order 1	0.41		-0.48		-0.52		-0.63	*
	Bacteria	Gemmatimonadetes	Gemmatimonadetes (class)	Unknown Gemmatimonadetes (class) order 2	0.72	***	-1.04	***	-0.79	***	-0.59	***
	Bacteria	Gemmatimonadetes	Gemmatimonadetes (class)	Unknown Gemmatimonadetes (class) order 3	0.39		-1.56	***	-1.29	***	-1.27	***
	Bacteria	Gemmatimonadetes	Gemmatimonadetes (class)	Unknown Gemmatimonadetes (class) order 4	0.01		-1.39	***	-1.85	***	-2.25	***
	Bacteria	Gemmatimonadetes	Longimicrobia	Longimicrobiales	0.41	*	0.73	***	0.95	***	0.92	***
	Bacteria	Gemmatimonadetes	S0134 terrestrial group	-	0.55	*	-1.88	***	-2.01	***	-2.11	***
	Bacteria	Halanaerobiales phylum	Halanaerobiales class	Halanaerobiales	0.21		-1.30	***	1.02	***	0.89	***
	Bacteria	Ignavibacteriae	Ignavibacteria	Ignavibacteriales	0.83	**	-1.53	***	-1.76	***	-2.05	***
	Bacteria	Lentisphaerae	Oligosphaeria	SS1-B-02-17	-0.08		0.85	***	1.06	***	1.05	***
	Bacteria	NC10	-	-	0.70	**	-2.00	***	-2.71	***	-2.80	***
	Bacteria	NC10	Ca. Methylomirabilis class	Ca. Methylomirabilis order	0.59	*	-1.87	***	-1.61	***	-2.17	***
	Bacteria	NC10	Unknown NC10 class 1	-	0.51	**	0.05		-0.35	*	-0.61	***
	Bacteria	Nitrospirae	Nitrospira	Nitrospirales	-0.24		-0.26		-0.12		-0.32	
	Bacteria	Nitrospirae	Nitrospira	Unknown Nitrospira order 1	-0.34		-0.56	**	0.25		-0.22	
	Bacteria	Planctomycetes	BD7-11	-	0.25		-1.04	***	-0.67	*	-1.11	***
	Bacteria	Planctomycetes	OM190	-	0.27		0.32		1.05	***	0.69	***
	Bacteria	Planctomycetes	Phycisphaerae	CCM11a	0.36		-1.85	***	-2.00	***	-2.27	***
	Bacteria	Planctomycetes	Phycisphaerae	CPla-3 termite group	0.23		-1.34	***	-1.11	***	-0.78	***
	Bacteria	Planctomycetes	Phycisphaerae	mle1-8	0.57		-1.34	***	-0.71	*	-0.93	**
	Bacteria	Planctomycetes	Phycisphaerae	Phycisphaerales	0.27		0.53	***	1.15	***	0.82	***
	Bacteria	Planctomycetes	Phycisphaerae	S-70	0.54		-1.30	**	-1.73	***	-1.66	***
	Bacteria	Planctomycetes	Phycisphaerae	Tepidisphaerales	0.00		-0.80	***	-1.54	***	-1.27	***
	Bacteria	Planctomycetes	Phycisphaerae	Unknown Phycisphaerae order 1	0.27		0.89	***	1.51	***	1.24	***
	Bacteria	Planctomycetes	Phycisphaerae	Unknown Phycisphaerae order 2	-0.13		2.85	***	3.73	***	3.99	***
	Bacteria	Planctomycetes	Pla3 lineage	-	0.51	*	-1.62	***	-1.94	***	-2.31	***
	Bacteria	Planctomycetes	Pla4 lineage	-	0.18		-2.56	***	-0.72	***	-0.89	***
	Bacteria	Planctomycetes	Planctomycetacia	Planctomycetales	-0.27		-0.04		-0.11		-0.24	
	Bacteria	Planctomycetes	Planctomycetacia	Unknown Planctomycetacia order 1	-0.28		-0.35		-0.07		-0.13	
	Bacteria	Planctomycetes	Planctomycetacia	Unknown Planctomycetacia order 2	-0.29		-1.08	***	-1.11	***	-1.08	***
	Bacteria	Planctomycetes	Planctomycetacia	Unknown Planctomycetacia order 3	0.00		2.10	***	-0.53	***	-1.20	***
	Bacteria	Planctomycetes	vadinHA49	-	-0.51		-0.05		-0.20		-0.65	

*	padj < 0.05
**	padj < 0.01
***	padj < 0.001

Supplementary Table 3 (part 3/4). DESeq analyses comparing alfalfa and control treatment at order level. Log2Fold changes > 0 and < 0 denote increases and decreases, respectively, in the alfalfa treatment compared to the control.

					T0		T1		T2		T4	
	Kingdom	Phylum	Class	Order	log2Fold Change	padj	log2Fold Change	padj	log2Fold Change	padj	log2Fold Change	padj
Bacteria (3/3)	Bacteria	Proteobacteria	Alphaproteobacteria	-	0.47		-0.39		-1.80	**	-2.90	***
	Bacteria	Proteobacteria	Alphaproteobacteria	4-Org1-14	0.19		-1.95	***	-2.00	***	-2.07	***
	Bacteria	Proteobacteria	Alphaproteobacteria	Caulobacterales	0.12		1.08	***	1.22	***	1.13	***
	Bacteria	Proteobacteria	Alphaproteobacteria	Parvularculales	0.25		0.80	**	0.83	**	0.97	***
	Bacteria	Proteobacteria	Alphaproteobacteria	Rhizobiales	0.51	**	0.21		0.18		0.22	
	Bacteria	Proteobacteria	Alphaproteobacteria	Rhodobacterales	0.21		-0.57		-1.71	***	-1.26	***
	Bacteria	Proteobacteria	Alphaproteobacteria	Rhodospirillales	0.19		-0.26	**	-0.35	***	-0.36	***
	Bacteria	Proteobacteria	Alphaproteobacteria	Rickettsiales	0.01		0.79	***	-0.18		-0.18	
	Bacteria	Proteobacteria	Alphaproteobacteria	Sneathiellales	0.38		-1.21	***	-1.27	***	-1.41	***
	Bacteria	Proteobacteria	Alphaproteobacteria	Sphingomonadales	0.69	***	1.19	***	0.65	***	0.51	***
	Bacteria	Proteobacteria	Alphaproteobacteria	Unknown Alphaproteobacteria order 1	0.69	***	-0.33	*	-0.83	***	-1.29	***
	Bacteria	Proteobacteria	Alphaproteobacteria	Unknown Alphaproteobacteria order 10	-0.05		0.40	*	0.09		0.36	
	Bacteria	Proteobacteria	Alphaproteobacteria	Unknown Alphaproteobacteria order 11	0.45		0.26		0.46		0.37	
	Bacteria	Proteobacteria	Alphaproteobacteria	Unknown Alphaproteobacteria order 2	0.38		1.54	***	1.48	***	1.19	***
	Bacteria	Proteobacteria	Alphaproteobacteria	Unknown Alphaproteobacteria order 3	0.66		2.14	***	2.05	***	2.95	***
	Bacteria	Proteobacteria	Alphaproteobacteria	Unknown Alphaproteobacteria order 4	0.43	*	-1.23	***	-1.04	***	-0.73	***
	Bacteria	Proteobacteria	Alphaproteobacteria	Unknown Alphaproteobacteria order 5	0.47		-0.20		-0.32		-0.38	
	Bacteria	Proteobacteria	Alphaproteobacteria	Unknown Alphaproteobacteria order 6	0.48		-1.36	***	-0.93	***	-0.93	***
	Bacteria	Proteobacteria	Alphaproteobacteria	Unknown Alphaproteobacteria order 7	0.42		-0.76	**	-1.54	***	-1.68	***
	Bacteria	Proteobacteria	Alphaproteobacteria	Unknown Alphaproteobacteria order 8	0.29		-0.42	**	-0.16		-0.35	**
	Bacteria	Proteobacteria	Alphaproteobacteria	Unknown Alphaproteobacteria order 9	0.14		2.75	***	2.65	***	2.89	***
	Bacteria	Proteobacteria	Betaproteobacteria	-	0.36		0.08		-0.46	**	-0.48	**
	Bacteria	Proteobacteria	Betaproteobacteria	B1-7BS	0.17		-0.94	***	-1.09	***	-1.31	***
	Bacteria	Proteobacteria	Betaproteobacteria	Burkholderiales	0.37		0.30		-0.57	**	-0.54	**
	Bacteria	Proteobacteria	Betaproteobacteria	Hydrogenophilales	0.11		0.15		-0.39		-0.35	
	Bacteria	Proteobacteria	Betaproteobacteria	Methylophilales	-0.40		0.94	***	0.94	***	0.84	***
	Bacteria	Proteobacteria	Betaproteobacteria	Neisseriales	0.07		0.11		-0.25		-0.01	
	Bacteria	Proteobacteria	Betaproteobacteria	Nitrosomonadales	0.33	**	-0.22	*	-0.34	**	-0.34	**
	Bacteria	Proteobacteria	Betaproteobacteria	Rhodocyclales	0.35		-0.37		-0.72	**	-0.85	***
	Bacteria	Proteobacteria	Betaproteobacteria	SC-I-84	0.28	*	-0.21		-0.56	***	-0.51	***
	Bacteria	Proteobacteria	Betaproteobacteria	TRA3-20	0.55	*	-0.83	***	-0.83	***	-1.00	***
	Bacteria	Proteobacteria	Betaproteobacteria	Unknown Betaproteobacteria order 1	0.49	*	-0.43	*	-0.10		-0.17	
	Bacteria	Proteobacteria	Betaproteobacteria	Unknown Betaproteobacteria order 2	0.23		-0.87	***	-1.21	***	-1.65	***
	Bacteria	Proteobacteria	Betaproteobacteria	Unknown Betaproteobacteria order 3	-0.02		-1.76	***	-2.49	***	-2.23	***
	Bacteria	Proteobacteria	Ca. Tenderia class	Ca. Tenderia order	0.59	*	0.67	**	0.49	*	0.42	
	Bacteria	Proteobacteria	Deltaproteobacteria	Bdellovibrionales	0.24		-0.04		0.36	*	0.71	***
	Bacteria	Proteobacteria	Deltaproteobacteria	Bradymonadales	0.00		2.24	***	0.93	***	0.95	***
	Bacteria	Proteobacteria	Deltaproteobacteria	Desulfuromonadales	0.22		1.12	***	0.25		0.17	
	Bacteria	Proteobacteria	Deltaproteobacteria	Myxococcales	0.21		0.93	***	0.45	***	0.15	
	Bacteria	Proteobacteria	Deltaproteobacteria	NB1-j	0.49	**	-0.07		0.35	*	0.12	
	Bacteria	Proteobacteria	Deltaproteobacteria	Oligoflexales	-0.10		-0.41		-0.35		-0.31	
	Bacteria	Proteobacteria	Deltaproteobacteria	Syntrophobacterales	0.11		-0.31		0.26		0.06	
	Bacteria	Proteobacteria	Deltaproteobacteria	Unknown Deltaproteobacteria order 1	0.41		-0.16		-0.71		-1.08	**
	Bacteria	Proteobacteria	Deltaproteobacteria	Unknown Deltaproteobacteria order 2	-0.12		-1.14	***	-1.32	***	-2.80	***
	Bacteria	Proteobacteria	Deltaproteobacteria	Unknown Deltaproteobacteria order 3	0.45		2.68	***	2.53	***	2.21	***
	Bacteria	Proteobacteria	Deltaproteobacteria	Unknown Deltaproteobacteria order 4	0.53	*	-0.11		0.57	**	0.77	***
	Bacteria	Proteobacteria	Deltaproteobacteria	Unknown Deltaproteobacteria order 5	-0.34		1.38	***	0.46		0.68	*
	Bacteria	Proteobacteria	Deltaproteobacteria	Unknown Deltaproteobacteria order 6	0.37		-2.37	***	-3.15	***	-3.05	***
	Bacteria	Proteobacteria	Gammaproteobacteria	-	0.56	**	-0.49	**	-0.31	*	-0.49	**
	Bacteria	Proteobacteria	Gammaproteobacteria	34P16	0.12		1.45	***	2.97	***	2.75	***
	Bacteria	Proteobacteria	Gammaproteobacteria	Acidiferrobacterales	0.25		-1.52	***	-1.60	***	-1.56	***
	Bacteria	Proteobacteria	Gammaproteobacteria	Arenicellales	0.19		0.04		-1.22	***	-2.58	***
	Bacteria	Proteobacteria	Gammaproteobacteria	BD72BR169	-0.42		-0.21		1.28	***	1.28	***
	Bacteria	Proteobacteria	Gammaproteobacteria	Cellvibrionales	0.51	*	6.37	***	1.59	***	0.46	*
	Bacteria	Proteobacteria	Gammaproteobacteria	Enterobacteriales	3.95	***	-0.69	*	-2.58	***	-2.79	***
	Bacteria	Proteobacteria	Gammaproteobacteria	HTA4	-0.26	*	0.66	***	1.20	***	1.48	***
	Bacteria	Proteobacteria	Gammaproteobacteria	KI89A clade	-0.08		-0.10		-0.20		0.21	
	Bacteria	Proteobacteria	Gammaproteobacteria	Legionellales	0.00		0.67	**	1.27	***	0.99	***
	Bacteria	Proteobacteria	Gammaproteobacteria	Oceanospirillales	-0.37		3.62	***	3.63	***	2.62	***
	Bacteria	Proteobacteria	Gammaproteobacteria	Pseudomonadales	0.52		0.21		-0.65	**	-0.65	**
	Bacteria	Proteobacteria	Gammaproteobacteria	PYR10d3	0.19		-1.30	***	-1.20	***	-1.26	***
	Bacteria	Proteobacteria	Gammaproteobacteria	Thiotrichales	0.38		-3.54	***	-3.09	***	-2.82	***
	Bacteria	Proteobacteria	Gammaproteobacteria	Unknown Gammaproteobacteria order 1	0.67	*	1.36	***	2.11	***	2.06	***
	Bacteria	Proteobacteria	Gammaproteobacteria	Unknown Gammaproteobacteria order 2	0.46		0.53		0.13		0.72	*
	Bacteria	Proteobacteria	Gammaproteobacteria	WN-HWB-116	0.45		3.46	***	3.60	***	2.72	***
	Bacteria	Proteobacteria	Gammaproteobacteria	X35	-0.17		0.51		0.56		1.13	***
	Bacteria	Proteobacteria	Gammaproteobacteria	Xanthomonadales	0.42	*	0.65	***	0.46	**	0.33	
	Bacteria	Proteobacteria	JTB23	-	0.03		1.36	***	0.01		0.39	
	Bacteria	Proteobacteria	SPOTSOCT00	5m83	0.42		-0.55	**	-0.45	*	-0.34	
	Bacteria	Proteobacteria	Unknown Proteobacteria class 1	-	0.39		-0.53		-0.60	*	-0.60	*
	Bacteria	SPAM	-	-	-0.21		-2.15	***	-2.34	***	-2.15	***
	Bacteria	Spirochaetes	Spirochaetia	Leptospiriales	-0.32		1.41	***	0.40		0.13	
	Bacteria	Spirochaetes	Spirochaetia	Spirochaetales	-0.21		1.00	**	0.20		-0.88	**
	Bacteria	Verrucomicrobia	Ca. Methylacidiphilum	-	-0.48		3.27	***	1.07	**	0.27	
	Bacteria	Verrucomicrobia	OPB35 soil group	-	-0.36	*	1.36	***	0.71	***	0.27	
	Bacteria	Verrucomicrobia	OPB35 soil group	Pedosphaera order	-0.25		0.58	**	0.09		-0.17	
	Bacteria	Verrucomicrobia	Opitutae	Opitutales	-0.14		2.38	***	1.50	***	0.88	***
	Bacteria	Verrucomicrobia	Spartobacteria	Chthoniobacterales	-0.71	***	1.96	***	1.21	***	1.40	***
	Bacteria	Verrucomicrobia	Verrucomicrobiae	Verrucomicrobiales	-0.02		2.96	***	2.67	***	2.68	***

*	padj < 0.05
**	padj < 0.01
***	padj < 0.001

Supplementary Table 3 (part 4/4). DESeq analyses comparing alfalfa and control treatment at order level. Log2Fold changes > 0 and < 0 denote increases and decreases, respectively, in the alfalfa treatment compared to the control.

					T0		T1		T2		T4	
	Kingdom	Phylum	Class	Order	log2Fold Change	padj	log2Fold Change	padj	log2Fold Change	padj	log2Fold Change	padj
Archaea	Archaea	Ca. Woesearchaeota	Pacearchaeota	-	-0.20		-2.85	***	-2.17	***	-2.33	***
	Archaea	Euryarchaeota	Methanomicrobia	Methanosarcinales	-0.41		-3.27	***	-6.31	***	-5.11	***
	Archaea	Thaumarchaeota	Ca. Allo-Thaumarchaeota	FHMa11 terrestrial group (Group 1.1c)	-0.14		-2.99	***	-1.81	***	-2.08	***
	Archaea	Thaumarchaeota	Nitrososphaeria	Ca. Nitrosopumilales	-0.61	*	-2.31	***	-0.77	***	-0.50	*
	Archaea	Thaumarchaeota	Nitrososphaeria	Nitrososphaerales	-0.19		-2.23	***	-1.50	***	-1.12	***
Fungi	Opisthokonta	Ascomycota	-	-	-0.24		1.35	***	1.60	***	1.82	***
	Opisthokonta	Ascomycota	Amb-18S-784	-	-0.46		1.14	***	0.85	**	1.24	***
	Opisthokonta	Ascomycota	Dothideomycetes	Pleosporales	0.12		1.29	***	1.82	***	2.10	***
	Opisthokonta	Ascomycota	Eurotiomycetes	Eurotiales	-0.66		2.58	***	3.81	***	3.60	***
	Opisthokonta	Ascomycota	Leotiomycetes	Helotiales	-0.32		1.42	***	1.39	***	1.70	***
	Opisthokonta	Ascomycota	Pezizomycetes	Pezizales	0.54		1.59	**	1.65	**	2.55	***
	Opisthokonta	Ascomycota	Sordariomycetes	-	0.02		2.07	***	2.26	***	2.56	***
	Opisthokonta	Ascomycota	Sordariomycetes	Hypocreales	-0.33		1.43	***	2.18	***	1.99	***
	Opisthokonta	Ascomycota	Sordariomycetes	Microascales	-1.19	*	4.83	***	4.53	***	5.26	***
	Opisthokonta	Ascomycota	Sordariomycetes	Sordariales	-0.37		4.25	***	4.52	***	4.44	***
	Opisthokonta	Basidiomycota	Agaricomycetes	-	-2.06	***	-1.42	***	-0.76		-1.42	***
	Opisthokonta	Basidiomycota	Tremellomycetes	-	-0.20		0.04		0.15		0.62	*
	Opisthokonta	Basidiomycota	Tremellomycetes	Tremellales	-0.39		0.88	*	0.96	**	1.40	***
	Opisthokonta	Chytridiomycota	Chytridiomycetes	Chytridiales	-1.25	*	1.29	*	1.43	**	1.85	***
	Opisthokonta	Glomeromycota	Glomeromycetes	Diversisporales	-1.46	***	0.83	*	1.14	**	1.28	***
	Opisthokonta	Glomeromycota	Glomeromycetes	Glomerales	-1.00		2.22	***	-0.53		1.86	***
	Opisthokonta	Mortirellales	Mortirellales class	Mortierellales	0.93	**	1.30	***	0.96	**	1.62	***
	Opisthokonta	Mucorales	Mucorales class	Mucorales	-1.23	***	1.61	***	0.63	*	0.59	
	SAR	Peronosporomycetes	Peronosporomycetes	Pythium order	-1.20	**	3.24	***	2.71	***	2.71	***
	Opisthokonta	Zoopagomycotina	Zoopagales class	Zoopagales	-1.35	***	-0.89	**	0.15		-0.18	
Micro-eukaryotes	Amoebozoa	Cavosteliida	-	-	-1.16	**	0.80	*	1.78	***	1.99	***
	Amoebozoa	Dictyostelia phylum	Dictyostelia class	Dictyostelia	-0.21		5.42	***	6.39	***	7.36	***
	Amoebozoa	Didymium phylum	Didymium class	Didymium order	0.41		-1.00		2.08	**	0.94	
	Amoebozoa	Discosea	Centramoebida	Acanthamoeba order	-1.41	***	1.29	***	1.88	***	1.92	***
	Amoebozoa	Gracilipodida	Filamoeba class	Filamoeba order	-1.25	*	2.26	***	3.02	***	3.61	***
	Amoebozoa	Gracilipodida	LEMD267	-	-1.20	*	1.08	*	1.85	***	1.06	*
	Amoebozoa	Ischnamoeba phylum	Ischnamoeba class	Ischnamoeba order	-0.98	**	1.58	***	0.92	**	1.15	***
	Amoebozoa	Lamproderma phylum	Lamproderma class	Lamproderma order	-2.35	**	-1.23		1.20		-0.65	
	Amoebozoa	LEMD255	-	-	-1.53	***	-1.37	***	-0.30		-0.34	
	Amoebozoa	LKM74	-	-	-1.58	***	-0.68	*	0.56		0.60	
	Amoebozoa	Lobosa	Discosea	Flabellinia	-1.03	**	2.44	***	3.89	***	3.88	***
	Amoebozoa	Lobosa	Discosea	Vannellida	-0.73	*	5.45	***	3.97	***	4.29	***
	Amoebozoa	Lobosa	Tubulinea	Arcellinida	-1.47	***	-0.14		1.50	***	1.19	**
	Amoebozoa	Lobosa	Tubulinea	Euamoebida	-1.99	***	1.45	***	1.20	***	1.00	**
	Amoebozoa	Lobosa	Tubulinea	Leptomyxida	-1.79	***	1.58	***	1.88	***	2.59	***
	Amoebozoa	Lobosa	Tubulinea	Unknown Tubulinea order 1	-1.49	***	-0.86	*	0.36		0.26	
	Amoebozoa	Lobosa	Tubulinea	Unknown Tubulinea order 2	-1.94	***	1.29	***	2.98	***	2.79	***
	Amoebozoa	Lobosa	Tubulinea	Unknown Tubulinea order 3	-2.61	***	0.89		1.04	*	0.88	
	Amoebozoa	Lobosa	Unknown Lobosa class 1	-	-1.65	***	1.27	**	2.25	***	1.89	***
	Amoebozoa	Schizoplasmodiida	-	-	-1.44	***	-0.79	*	0.77	*	0.76	*
	Amoebozoa	Stemonitis phylum	Stemonitis class	Stemonitis order	-0.82		-1.85	***	-0.44		-0.63	
	Amoebozoa	Unknown Amoebozoa phylum 1	-	-	-1.34	***	-0.43		-0.54		-0.57	
	Amoebozoa	Unknown Amoebozoa phylum 2	-	-	-2.55	***	2.66	***	2.91	***	3.45	***
	Amoebozoa	Unknown Amoebozoa phylum 3	-	-	-1.52	***	1.14	**	1.35	***	1.30	***
	Apusozoa superkingdom i	Apusozoa	-	-	-1.55	***	0.68		1.25	***	1.00	**
	Apusozoa superkingdom i	Apusozoa	Rigifilida class	Rigifilida	-1.63	***	0.56		-1.47	***	-1.03	**
	Excavata	Heterolobosea	Tetramitia	Tetramitus order	-1.73	**	0.87		1.45	**	1.23	*
	Excavata	Heterolobosea	Tetramitia	Vahlkampfia order	-1.36	*	2.79	***	3.41	***	3.52	***
	Excavata	Jakobida	Unknown Jakobida class 1	-	-1.67	***	0.65	*	1.38	***	1.31	***
	Excavata	Kinetoplastea	Metakinetoplastina	Neobodonida	-1.79	**	1.15	*	1.32	*	1.15	*
	Excavata	Kinetoplastea	Unknown Kinetoplastea class 1	-	-1.69	*	0.09		1.63	**	1.06	
	Opisthokonta	Nematoda	Chromadorea	Araeolaimida	-1.08		7.26	***	7.09	***	5.49	***
	Opisthokonta	Nematoda	Chromadorea	Rhabditida	-0.85		2.68	**	1.77		3.73	***
	Opisthokonta	Nematoda	Chromadorea	Tylenchida	0.95		-1.44		-1.91	*	-0.62	
	Opisthokonta	Nucleariida	Nucleariidae class	Nucleariidae order	-0.53		4.74	***	5.68	***	5.26	***
	SAR	Unknown	-	-	-0.88	**	0.35		2.34	***	2.61	***
	SAR	Bicosoecida	Bicosoecida class	Bicosoecida	-1.03	***	0.60	*	0.90	***	1.42	***
	SAR	Cercozoa	-	-	-1.15	**	0.33		0.52		0.12	
	SAR	Cercozoa	Cercomonadidae class	Cercomonadidae order	-1.27	***	1.48	***	2.18	***	2.02	***
	SAR	Cercozoa	Cercozoa	Gymnophrys order	-1.48	***	0.13		1.26	***	2.00	***
	SAR	Cercozoa	Glissomonadida class	Glissomonadida	-1.29	***	1.04	***	2.60	***	2.92	***
	SAR	Cercozoa	Imbricatea	Euglyphida	-1.24	***	0.57		0.88	**	0.48	
	SAR	Cercozoa	Imbricatea	Nudifila order	-2.70	***	0.52		0.69		-0.22	
	SAR	Cercozoa	Imbricatea	Thaumatomonadida	-1.31	***	1.91	***	1.40	***	0.54	
	SAR	Cercozoa	Metromonadea	Metopion order	-0.10		0.54		0.37		-0.03	
	SAR	Cercozoa	Metromonadea	Unknown Metromonadea order 1	-0.70		0.20		1.28	***	1.70	***
	SAR	Cercozoa	Novel Clade Gran-5	-	-1.23	**	1.62	***	2.90	***	3.00	***
	SAR	Cercozoa	Thecofilosea	-	3.34	***	0.88	***	1.65	***	1.62	***
	SAR	Cercozoa	Thecofilosea	Amb-18S-431	-1.61	***	1.17	***	1.61	***	1.61	***
	SAR	Cercozoa	Thecofilosea	Cryomonadida	-1.34	***	2.62	***	3.50	***	3.41	***
	SAR	Cercozoa	Unknown Cercozoa class 1	-	-1.03		2.80	***	4.42	***	4.20	***
	SAR	Chrysophyceae phylum	Chrysophyceae	Amb-18S-462	-0.96	**	3.29	***	3.24	***	4.09	***
	SAR	Ciliophora	Colpodea	Platyophryida	0.19		0.37		-0.29		-1.13	
	SAR	Ciliophora	Unknown Ciliophora class 1	-	-0.60		2.38	***	0.75	*	0.50	
	SAR	Labyrinthulomycetes	Labyrinthulomycetes	Sorodiplophrys order	-1.21	*	2.19	***	2.76	***	2.93	***
	SAR	Labyrinthulomycetes	Labyrinthulomycetes	Unknown Labyrinthulomycetes order 1	-1.45	***	2.73	***	3.18	***	3.65	***
	SAR	Labyrinthulomycetes	Labyrinthulomycetes	Unknown Labyrinthulomycetes order 2	0.74		2.76	***	3.43	***	4.08	***
	SAR	Labyrinthulomycetes	Labyrinthulomycetes	Unknown Labyrinthulomycetes order 3	-0.92	**	1.22	***	3.37	***	3.54	***
	SAR	Unknown Rhizaria phylum 1	-	-	-1.65	**	-1.57	**	-0.57		0.19	
	SAR	Xanthophyceae	Mischococcales class	Mischococcales	-1.67	***	0.00		1.12	***	1.66	***

*	padj < 0.05
**	padj < 0.01
***	padj < 0.001

Supplementary Figure S1

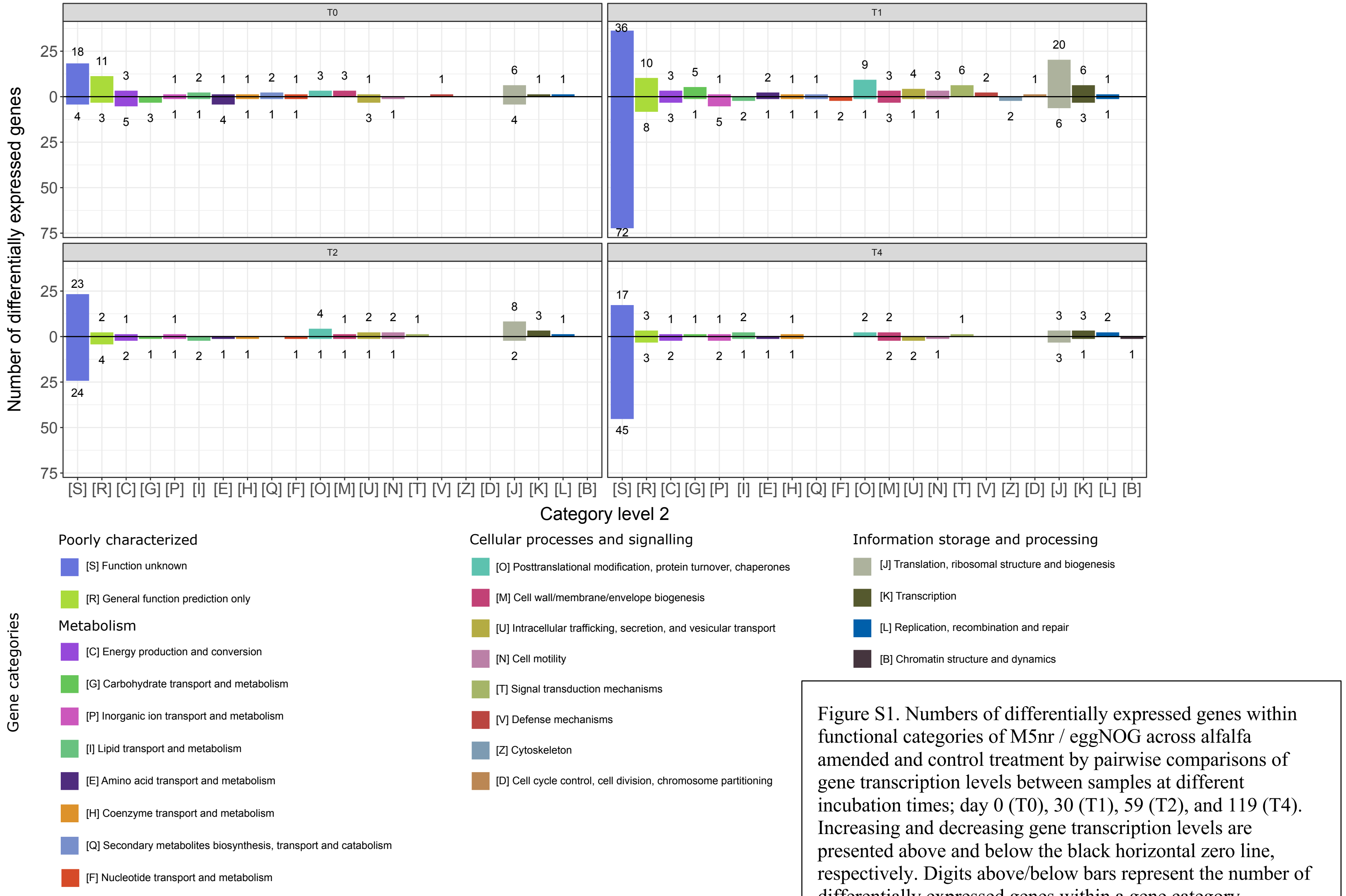

Figure S1. Numbers of differentially expressed genes within functional categories of M5nr / eggNOG across alfalfa amended and control treatment by pairwise comparisons of gene transcription levels between samples at different incubation times; day 0 (T0), 30 (T1), 59 (T2), and 119 (T4). Increasing and decreasing gene transcription levels are presented above and below the black horizontal zero line, respectively. Digits above/below bars represent the number of differentially expressed genes within a gene category

Supplementary Figure S2

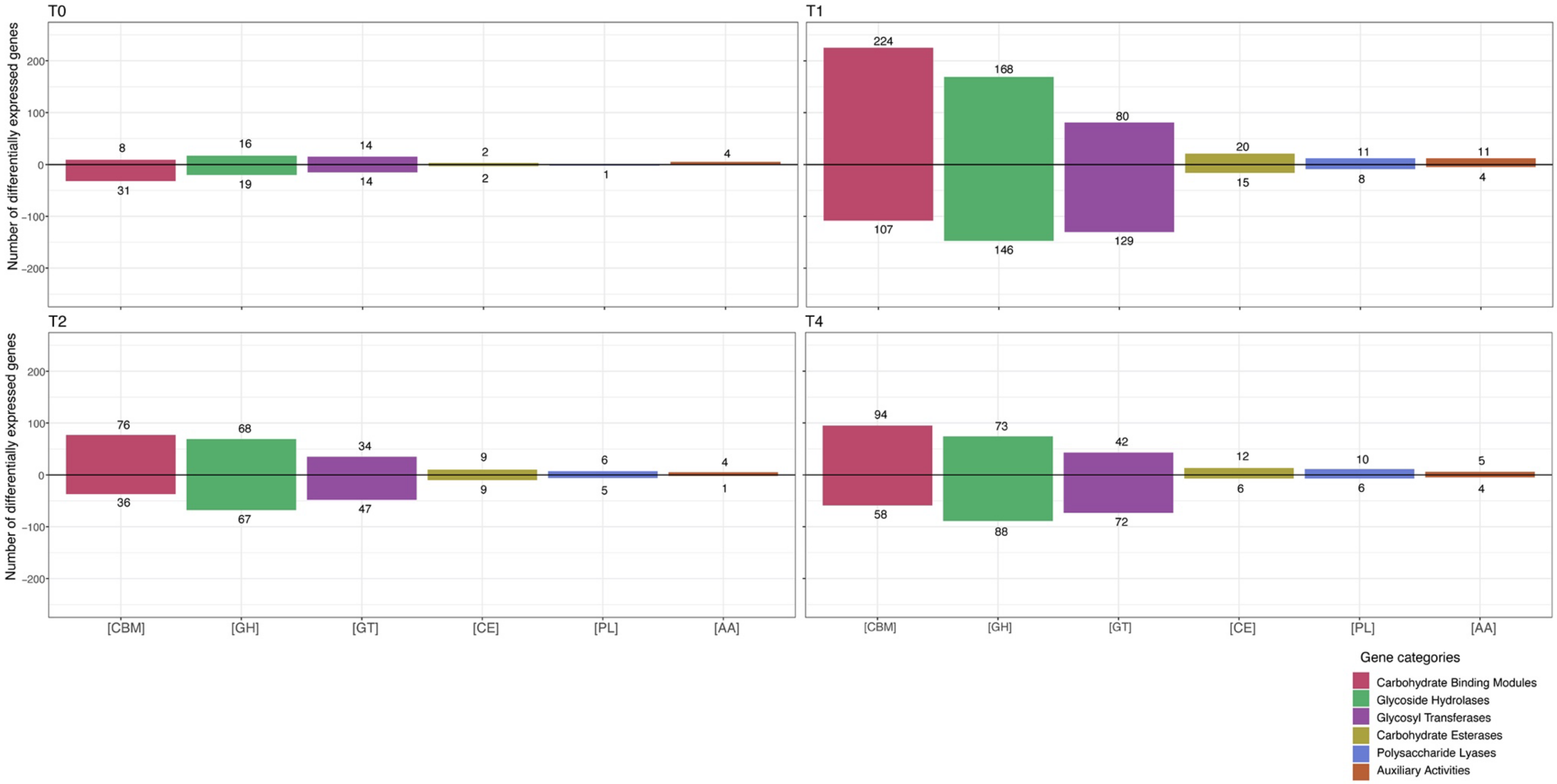

Figure S2. Numbers of differentially expressed genes within functional categories of CAZy across alfalfa amended and control treatment by pairwise comparisons of gene transcription levels between samples at different incubation times; day 0 (T0), 30 (T1), 59 (T2), and 119 (T4). Increasing and decreasing gene transcription levels are presented above and below the black horizontal zero line, respectively. Digits above/below bars represent the number of differentially expressed genes within a gene category.

Supplementary Figure S3

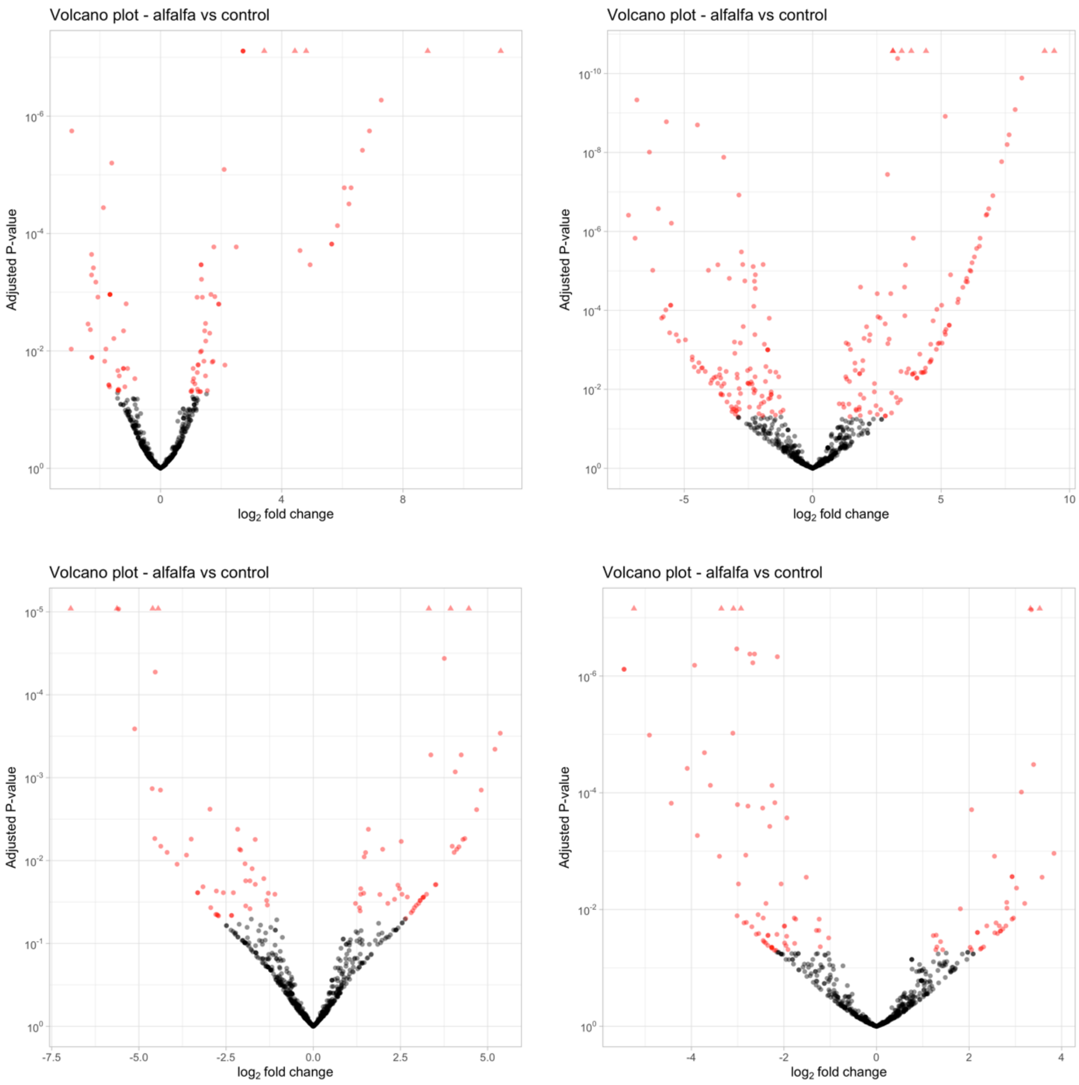

Figure S3. Volcano plots from the SARTOOLS analyses of mRNA annotated by the M5nr / eggNOG across alfalfa amended and control treatment by pairwise comparisons of gene transcription levels between samples at different incubation times; day 0 (T0; top left), 30 (T1; top right), 59 (T2; bottom left), and 119 (T4; bottom right).

Supplementary Figure S4

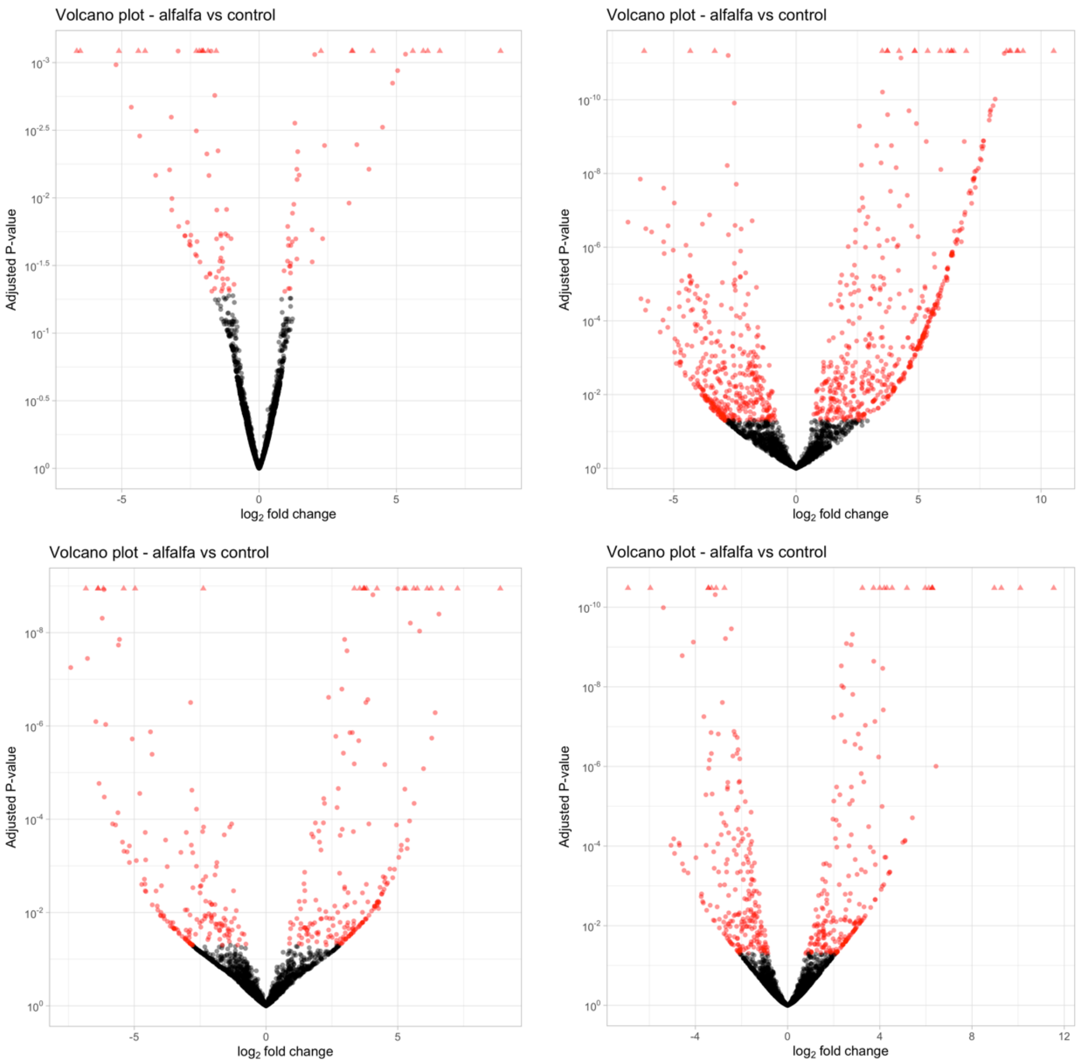

Figure S3. Volcano plots from the SARTOOLS analyses of mRNA annotated by CAZy across alfalfa amended and control treatment by pairwise comparisons of gene transcription levels between samples at different incubation times; day 0 (T0; top left), 30 (T1; top right), 59 (T2; bottom left), and 119 (T4; bottom right).
